# Supplementary material for: Solid waste management practices and challenges in Besisahar municipality, Nepal
Source: PLoS One. 2024 Mar 21;19(3):e0292758. doi: 10.1371/journal.pone.0292758 (PMC10956786; doi:10.1371/journal.pone.0292758)
Supplement: S1 File — (DOCX) [file pone.0292758.s001.docx]

**Survey questionnaire (English version)**

Dear participants,

The purpose of this survey is to collect valuable insights regarding the comprehension, perspectives, and implementation of household and municipal solid waste management practices within Besisahar municipality, Nepal. Your honest and precise responses will significantly enrich the calibre of our research. Be assured that any data you supply will be kept entirely confidential and exclusively employed for the designated research objectives. Your involvement has no personal ramifications, ensuring absolute anonymity. To assist you in participating, precise guidelines are furnished for the forthcoming questions.

**General Information:**

Your name is not required for this questionnaire.

Please use a "√" to mark the designated boxes.

Circle the letter that corresponds to your answer for multiple-choice questions.

When responding to open-ended questions, kindly provide pertinent information within the provided space.

It is important to complete the questionnaire on your own, without seeking input from your friends. Your individual perspectives are valuable to us.

**Respondent Information**

1. Gender

2. Age

3. Educational Level

5. Duration of Residency in the Municipality

6. Occupation

**Primary Inquiries Presented in the Survey**

**Part-I: Waste Generation**

1. On average, how much household waste do you estimate your family generates in a day?

2. Can you categorize the types of waste typically produced in your household

3. What are the main sources of waste in your household?

4. Are there specific items that contribute significantly to the waste your household generates?

5. How has your household's waste generation changed over the past few years? Have you noticed any trends?

**Part-II: Waste Segregation**

1. Are you familiar with the concept of waste segregation at the source?

2. Do you currently practice waste segregation at home? If yes, could you describe how you separate different types of waste?

3. What factors influence your decision to segregate waste or not?

4. Are there any challenges you face in properly segregating waste at home?

5. Are you aware of the benefits of waste segregation for recycling and proper disposal?

6. Do you know the specific guidelines for segregating waste in your community or locality?

7. How do you dispose of segregated waste?

8. Are there designated collection points for different types of waste in your area?

9. Have you noticed any improvements in waste management or collection since you started segregating waste?

**Part-III: Attitudes and Practices**

1. How important do you think proper waste segregation is for environmental protection and sustainability?

2. Do you believe that individual efforts in waste segregation can make a significant impact on the overall waste management process?

3. Are you motivated to improve your waste segregation practices? If yes, what drives your motivation?

4. Have you participated in any community initiatives, workshops, or campaigns related to waste segregation?

5. Would you be interested in learning more about effective waste segregation methods or participating in educational programs?

**Part-IV: Knowledge of Households towards Solid Waste Management**

1. What does the term "solid waste management" mean to you?

2. Can you describe the different stages of the solid waste management process?

3. How familiar are you with the concept of waste reduction and its importance in waste management?

4. Do you know the various methods of waste disposal, including landfilling, incineration, and recycling?

5. Are you aware of the potential environmental and health impacts of improper waste disposal?

6. Can you identify some common materials?

7. Are you aware of composting organic waste at the household level?

8. Have you heard of the concept of "reduce, reuse, recycle"?

9. Are you knowledgeable about any local regulations or guidelines related to waste sorting, collection, and disposal?

10. How do you separate different types of waste at home before disposal?

11. Do you know where your household waste goes after it's collected?

12. Are you aware of the waste management facilities in your area?

13. Have you ever participated in community clean-up drives or awareness programs about solid waste management?

14. Are there any challenges you face in practicing proper waste management at home?

15. How do you think your individual actions regarding waste management contribute to the overall cleanliness of your community?

16. Are you interested in learning more about waste management practices and how to improve them?

**Part-V: Generation of Institutional, Commercial, and Industrial Waste**

***Institutional Waste:***

1. Can you briefly describe the type of institution you are associated with?

2. What are the primary activities carried out in your institution that generate waste?

3. How does your institution manage different types of waste, such as paper, plastic, and hazardous materials?

4. Are there any specific waste reduction or recycling initiatives in place within your institution?

5. Do you believe that your institution's waste management practices align with environmental sustainability goals?

***Commercial Waste:***

1. Could you provide a general overview of your business or commercial establishment?

2. What types of products or services does your business offer, and how do these activities contribute to waste generation?

3. How does your business currently handle waste disposal and management?

4. Are there any efforts or strategies in place to minimize waste generation within your business operations?

5. Are you aware of any regulations or guidelines specific to commercial waste management that your business follows?

***Industrial Waste:***

1. Can you describe the industrial processes that take place within your facility?

2. What are the main types of waste generated as a result of these industrial processes?

3. How does your industry ensure the proper disposal and management of hazardous or non-hazardous waste?

4. Are there any technologies or practices your industry employs to reduce waste generation or promote recycling?

5. Have there been any notable changes in your industry's waste management practices in recent years?

***General Questions:***

1. How does waste generation in your institution/business/industry compare to previous years?

2. Are there any challenges you face in effectively managing and reducing waste generation in your context?

3. Are you aware of the environmental impacts associated with improper disposal of institutional, commercial, or industrial waste?

4. Have you implemented any specific measures to raise awareness among employees/staff about responsible waste management?

5. Are there any future plans or goals to improve waste management practices in your institution/business/industry?

**Part-VI: Response regarding waste disposal in BM**

Please, choose one of the alternatives by putting “√” in the given tables.

| SN | Questions | A | B | C | D | E |
| --- | --- | --- | --- | --- | --- | --- |
| 1 | Suitable for dumping areas on the riverbank |  |  |  |  |  |
| 2 | Impact of dumping on river banks |  |  |  |  |  |
| 3 | Segregation of waste before dumping |  |  |  |  |  |
| 4 | Waste treatment before dumping |  |  |  |  |  |
| 5 | Private waste collector activities on dumping |  |  |  |  |  |
| 6 | Municipality in waste disposal activities |  |  |  |  |  |
| 7 | Implementation of SDGs |  |  |  |  |  |

Note: A: Strongly agree; B: Agree; C: Disagree; D: Strongly disagree; E: No knowledge

Do you have any additional ideas or perspectives that have not been discussed previously?

Thank you!
