# Supplementary material for: Solid waste management practices and challenges in Besisahar municipality, Nepal
Source: PLoS One. 2024 Mar 21;19(3):e0292758. doi: 10.1371/journal.pone.0292758 (PMC10956786; doi:10.1371/journal.pone.0292758)
Supplement: Attachment — Submitted filename: PONE-D-23-04289.pdf [file pone.0292758.s002.pdf]

Manuscript Click here to access/download;Manuscript;Manuscript.docx

Solid waste management practices and challenges in Besisahar municipality, Nepal

Mahendra Aryal[1] and Sanju Adhikary[2]

[1]Department of Chemistry, Tribhuvan University, Tri-Chandra Campus, Kathmandu, 44600, Nepal.

[2]Department of Economics, School of Law, Kathmandu University, Dhulikhel, Nepal.

Corresponding Author:

Asst. Prof. Sanju Adhikary

Department of Economics, School of Law, Kathmandu University, Dhulikhel, 45200, Nepal

Email: sanju.adhikary@ku.edu.np

Tel: 00977-9860365137

Abstract

This study is a comprehensive assessment of the waste management system in Besisahar municipality. Information and some data have been collected from the municipality of Besisahar, followed by interviews with municipal officials responsible for waste management, stakeholders, waste workers, and residents. A total of 230 households, 20 schools, 10 government and private offices, 10 financial institutions, 60 commercial hotels, restaurants, and shops, and 20 medical shops and healthcare institutions, were selected in this study by random sampling. An extensive field study was conducted within all municipal wards and at dump sites. The results indicated that 42.14 % of solid waste was collected through door-to-door collection services, 5.87% was mismanaged in open public places, 11.21% was used as compost manure, and the rest was discarded on river banks, dug up, and burned. A large component of the characterization of household waste consisted of organic waste (68.03%), followed by paper/paper products(8 .13%), agricultural waste (5.5%), and plastic (5.21%) in the Besishahar municipality. Solid waste generation was found to be at 197.604 g/capita/day, as revealed by cluster sampling in 230 households. Around 4.285 tons-solid waste/day were generated in urban areas, while 6.887 tons-solid waste/day was estimated for the whole municipality. Currently, solid waste is dumped on river banks, open fields, and springs, creating environmental and health hazards. The findings of this study will be useful to Besisahar municipality and its stakeholders in forming policies that facilitate waste management practices in this region and promote sustainable waste management systems.

Keywords: Nepal, Household; Waste management, Environmental pollution, Health, SDGs

Introduction

Minimising solid waste management (SWM) has become a global challenge due to limited resources, an increasing population, and rapid urbanization and industrialization [1]. In developing countries, most waste is composed of organic materials, which are generally three times higher than in industrialized countries [2]. The improper disposal of e-waste, polythene materials, and medical waste in

the locality also contributes to pollution and public health hazards [3,4,5]. Waste management (WM) involves several interdependent activities that are coordinated and controlled efficiently. Among these activities are generation, storage, collection, transportation, recycling, and disposal [6]. Moreover, waste recycling reduces greenhouse gas emissions and particulates from incinerators, and increases the lifespan of landfills [7,8].

The Solid Waste Management Act of 2011 was adopted by Nepal on June 15, 2011. It aims at keeping the environment clean and healthy by minimising the adverse impacts of solid waste on public health and the environment [9,10]. According to the Act, local authorities are required to promote recycling, reuse, and reduce municipal solid waste (MSW), in addition to segregating it at its source [11,12]. Also, the Act permits the imposition and collection of service fees against solid WM services and specifies principles for fixing such charges and procedures for collecting and utilizing them [9]. Solid waste collection, treatment, and final disposal are all the responsibility of Nepalese municipalities [1]. Moreover, competitive bidding allows non-public organizations, community-based organizations, and non-government organizations to be involved in SWM [9].

Sustainable Development Goals (SDGs) were established as a result of the UN Conference on Sustainable Development in June 2012 and the UN General Assembly in September 2014. In the SDGs, 17 goals were set up for reducing poverty, enhancing social equality, decreasing pollution levels, and making cities more livable [13]. To achieve sustainability, the Global Waste Management Goals include ensuring affordable, secure, and accessible solid waste collection services; preventing open burning and dumping; and managing environmentally sound WM by 2030 [14,15]. Nepal is committed to the current global initiative as a member of the UN [16].

An integrated and sustainable WM approach is urgently required in Besisahar municipality (BM) to protect human and environmental health. The purpose of this study is to investigate the scenarios regarding SWM practices in rural and urban areas of BM and to achieve sustainable WM. This study would help both local communities and BM in managing waste.

Methodology

Study area

Besisahar is the headquarter of Lamjung district, and the study area covers the village and urban areas with dense populations. There are 11 political wards in BM, covering 127.64 km^2 of geographical area. It lies between latitude 28° 23' 26.41'' N and longitude 84° 34' 28.13'' E. The elevation ranges from 728 to 1268 meters above sea level, with a mean annual temperature of 25 ^{O}C. The geographical map of BM is given in Fig. 1.

Population of BM

BM consists of high-, medium-, and low-density residential areas. Several government offices, schools, colleges, hospitals, industries, hotels, agricultural farms, non-governmental organizations, and religious and cultural landmarks are located in the BM. A total of 44,598 people lived in BM, including 22731 males and 21867 females. Out of the total wards, the highest number of residents was 8703 in ward number 7, while the fewest number of residents was 1375 in ward number 4. There were 9834 households in BM. Ward 8 had the highest number of households with a total of 1994, and ward 4 had the lowest with just 247 households (Table 1). According to the BMP [17], the majority of residents in BM were between the ages of 10 and 14, whereas the least populated age group was between 70 and 74.

Research Method

To conduct the present study, both primary and secondary sources of information were used. Observations, interviews, and questionnaire surveys were conducted during January–May 2022 AD. Primary and secondary data were obtained through interviews with various officials of the BM of all political wards, WM employees, residents of the study areas, academic and commercial establishments, as well as relevant stakeholders.

A cluster sampling technique was used to collect data from 230 households. The questionnaires were prepared about types of waste and their composition, collection, and management. Participants were asked to describe their socioeconomic status, the types of waste generated by their households, resource

management, as well as how they stored and disposed of their solid waste. About 52.17% of the sample size (120) represented rural households located in wards 4, 5, 6, 9, 10, and 11, 26.086% of the sample size (60) represented semi-urban households located in wards 1, 2, and 3, while 21.739% of the sample size (50) represented urban households located in wards 7, and 8, respectively. Each cluster consisted of ten households. Twenty-three clusters were examined in total. As part of the study, 20 schools, 10 government and private offices, 10 financial institutions, 60 commercial hotels, restaurants, and shops, as well as 10 medical shops and 10 healthcare institutions, were selected through a random sampling method. There were separate questionnaires prepared for the survey of households and the survey of the institutional and commercial sectors, as well as for the municipality office to collect data related to SWM.

Determination of waste

To educate households, hotels, restaurants, shops, government and non-government offices, schools, and hospitals about the importance of collecting waste within 24 hours, pre-visits were conducted at selected locations and the waste collection bags were provided to them. Solid wastes were then segregated into organic wastes, plastics, paper products, metals, glass, rubber, textiles, construction debris, hazardous wastes, and other wastes. The amounts of different wastes collected from homes, hotels, hospitals, schools, factories, offices, and governmental and private organizations were measured on a wet weight basis with a digital balance.

Data analysis

General data were analysed using descriptive statistics. The analysis of the data was carried out using both quantitative and qualitative methods. Interviews and observation frames yielded qualitative information that was coded, classified, and analyzed into different themes. Microsoft Excel was used to analyze the data.

Results and discussion

Waste generation and segregation

It is necessary to handle, store, collect and dispose of waste generated by human activities to reduce the risk of environmental and public health problems [19]. As shown in Table 2, the solid wastes generated in the rural and urban areas of BM were categorized based on their composition. The most prevalent waste types were found to be food, paper, plastic, rubber, wood, metals, glass, hazardous, biomedical, agricultural, and industrial wastes; however, due to socioeconomic factors and cultural practices, the types and rates of generation of waste vary greatly from place to place [20]. As seen in Fig. 2, different types of waste were transported to the nearby collection centre for urban areas. Due to a direct economic benefit, some residents separate some kinds of waste, including paper and metals. The results of the questionnaire study revealed that 72% of household respondents knew about waste separation, but they did not apply it practically, whereas only 28 % of respondents indicated that they were utilizing such knowledge to separate waste properly at the household level.

Household SWM practices

Open dumping in public places or along the banks of rivers or streams is a traditional method of handling solid waste in the BM. An overview of household SWM in BM is provided in Table 3 [17]. Many village households had their dumping sites where they made compost and only a small amount of waste was disposed of outside. It was observed that some households in villages disposed of waste near public places. A large number of households did not separate their waste when dumping it. Households even threw garbage into urban areas without respecting the collection centres. Several households and individuals were observed to be less aware of where to dump their waste. It was reported that solid waste collection was implemented in wards numbers 1, 2, 3, 7, 8 and 9. As of now, there is no waste collection service in wards 4, 5, 6, 10, and 11. In addition, wards no. 7, 8, and 9 of urban areas had a door-to-door solid waste collection service, which was found to be at 87.8, 94.4, and 10.3%. Ward no. 5 had the lowest rate of digging and burning solid waste and residents managed in their compounds (3.4%), while ward no. 8 had the highest rate (80.2%). The proportion of waste managed by households and sent to waste collection centres varied from 3.4% in ward no. 7 to 60.6% in ward no. 10. The highest rate of waste dumped in nearby spring-fed rivers or channel runoff was found at 64.4% in ward no. 5, and the lowest was at 0.2% in ward no. 8. For

the use of compost manure, a minimum and maximum range was determined at 0.4 and 49.3% in wards no. 4 and 2, while for mismanagement in public places, 0 and 37.6% were determined in wards 7 and 9, respectively.

Table 4 shows household SWM with total family number and percentage in BM [17]. Among households, only 42.14% had door-to-door household waste collection services, while 5.87% had mismanaged their solid waste in public places. In addition, only 11.21% of solid wastes were used as compost manure, with the majority of the rest being discarded on river banks, in public places, dug up, and burned. Household wastes were seen being dumped into bodies of water directly, which can harm the environment and the general public's health. The overall results indicated that the WM system of the BM was ineffective and unscientific. WM practices should be implemented in such a way that the result of waste in the external environment is minimized [11]. The segregation of these wastes by waste generators is a necessary component of a successful SWM system, but it is not done in the BM.

Households waste characterization

Most of the food waste generated in villages was used as animal feed, and organic waste was relatively low in comparison to urban areas. The waste generation, including paper, plastic, rubber, electronics, pharmaceuticals, etc., was much higher in urban areas compared to villages. Over the past few years, there has been an increase in the amount of plastic waste. During the field survey, electronic, and hazardous waste were observed in public places, and it showed that people in the urban and village areas have less awareness regarding the categories of waste settlement. It was noticed that neither the municipality nor the private waste collectors characterize waste, and neither has a system to determine how much waste has been generated and collected.

The composition of household solid waste generated per day in BM and the outcomes are summarized in Fig. 3. The results showed that almost all of the waste generated by households was organic (68.03%). The results further revealed that besides organic wastes, 5.5% of the wastes were agricultural wastes, 3.81% construction debris, 5.21% plastics, 1.001% glass, 8.13% paper, 8.13% textiles, 0.54% metals, 0.1% rubber, 0.1% pharmaceuticals, 0.5% electronics, and 4.8% other wastes. The composition of

household waste in Jeetpur Simara Sub-Metropolitan City was estimated to be 80% organic, 10% plastic, 6% paper, and 4% inert [21]. Among 53 municipalities in Nepal, organic waste had the highest household waste composition at 66%, followed by plastics at 12%, and paper products and paper waste at 9% [9]. An investigation conducted in 1995 found that only 38% of the farmers buried or burned old or unused pesticides, whereas others were disposed of in waste containers as solid waste in open fields [10].

Household waste generation

To determine the number of grams/capita/day of waste from each household, the total weight of waste from 230 household samples was divided by the number of people living in each household. It was reported that the average family size for BM was 3.78 members [17]. By comparison, Dangi et al. [11] found a family size of 5.2 in Tulsipur, Dang, and Dangi et al. [22] found a family size of 5.7 in Kathmandu city. According to an ADB [9] study conducted in 53 different municipalities in Nepal, the average number of family members in each family ranged between 5 and 6. In our study, the value of family size is lower than the values reported elsewhere [9,11,22].

The solid waste generation rate in different wards of BM is given in Fig. 4. There was a significant amount of waste generated from urban households, including glass bottles, plastic, papers, rubber, metals, medicines, wood, electronics, tin, cans, construction waste, packaging materials, and hazardous waste. Compared to urban households, rural households generated mainly plastics, agricultural wastes, papers, fertilizers, some metals, and pesticides, which can be attributed to the low waste generation. The minimum and maximum average waste generation of 1.64 and 9110 g/capita/day were observed from 230 household samples. The average amount of household waste generated was calculated to be 197.604 g/capita/day for the BM. Based on the total of 3300 households from 60 municipalities with 55 households each, an average per capita household waste generation of 115 g/day was estimated by Pathak et al. [20]. The household survey in Jeetpur Simara Sub-Metropolitan City found an average household waste generation rate of 120 g/capita/day [21]. For the Tulsipur municipality, Dangi et al. [11] determined an average of 330 g/capita/day. According to ADB [9], the household waste generation rate in Nepal's 53 municipalities ranged from 75 g/capita/day to 278 g/capita/day in Triyuga and Inaruwa municipalities. The average

household waste generation rate in the Triyuga municipality of Nepal was 317 g/capita/day. Possible explanations for the slightly different values include the different sample sizes taken in rural and urban areas.

A total of 34,857 people (78.16%) were permanently residing in BM. Furthermore, 5506 people (12.35%) of the BM lived in other parts of Nepal, while 4235 people (9.50%) were abroad. It was reported that 3708 households (35.58%) had at least one member living outside the family [17, 18]. The amount of household solid waste produced in BM was 6.887 tons/day, which was obtained by multiplying the average per capita waste generation rate by the total population minus absentees in the municipality according to the 2021 census. The total household solid waste generation was calculated at 14.2 tons/day in Tulsipur municipality in Dang, Nepal [11].

An analysis of skewness and kurtosis was used to determine if the data gain was normally distributed or not. Statistics for solid waste analysis calculated from 230 households are shown in Table 5. The calculated coefficients of skewness and kurtosis were found to be 7.290 and 58.350, respectively. In general, a normal distribution of waste generation has coefficients of skewness and kurtosis of 0 and 3, respectively [23, 24]. As a result of a positive skewness and a slightly higher kurtosis in this study, the distribution of waste in households appears to be random. There is a significant difference between the median and mean values for household waste generation. Hence, the standard deviation of 923.69 indicates that the data is most closely distributed around its mean waste generation value. The results showed that the majority of households produced a lower generation rate, whereas the minority of households had a higher mean generation rate. It was also observed that larger families resulted in a higher household waste generation rate with a lower per capita waste generation rate [11,20].

Degree of urbanization

The degree of urbanization may have a significant effect on per capita waste generation. As shown in Table 6, rural, semi-urban, and urban areas generate different amounts of waste. It is noted that household waste generation rates vary according to urbanization. The study found that urban households generated the highest amount of waste at 450.018 g/household/day, followed by semi-urban households at 345.44

g/household/day, and rural households at 35.771 g/household/day. Accordingly, the larger the population, the higher the amount of household waste generated per day. Han et al. [25] reported that the degree of urbanization was positively correlated with MSW generation in China.

Correlation between households economy and waste generation

There were 1,772 establishments involved in various economic activities in BM, as reported in the 2018 Economic Census. The establishments employed 12,466 individuals, of which 6,572 were males, and 5,894 were females, as either self-employed or employees. An average of 7.03 people were employed in each business, with males accounting for 3.71 and females accounting for 3.33, respectively. The ratio of males to females engaged in economic activities in establishments was 1.12 [26].

Most households with the highest incomes reside in the urban areas of wards 7 and 8. A majority of households in wards 1,2, 3, 4, 5, 6, 9, 10 and 11 were in villages and has low incomes. In addition, some households of wards 1, 2, 3, and 9 were semi-urban and they have middle incomes. There were 741 households with a very low income (7.54%) a total of 9834 households. The majority of 41.21% of households were lower-middle-income, whereas 35.73% of the households were middle-income. Higher-income households were represented by 10.9%, while the highest-income household made up 4.62%. In this study, income is positively correlated to waste generation rate.

Table 7 presents the average household income/expenditures in BM. There were differences in household waste generation rates based on incomes and expenditures. Household expenditure capacity had a significant impact on waste generation. In Fig. 5, it is shown that households with higher average expenditures generated more waste per day. A household with a monthly expenditure of at least US$ 767 generated approximately 950 g/household/day, whereas a household with a monthly expenditure of less than US$ 38 generated an average of 216 g/household/day. The results of this study agree with those of ADB [9] and Pathak et al. [20] in many municipalities in Nepal.

Generation of institutional and commercial waste

As part of the study, the average composition of institutional and commercial waste was obtained from the analysis of waste samples. There was an average waste generation rate of 7.81 kg/day for hospitals,

3.27 kg/day for hotels and restaurants, 2.5 kg/day for commercial shops, 1.67 kg/day for academic institutions, 0.43 kg/day for banks and financial institutions, and 0.32 kg/day for government and private offices. The waste composition analysis in all the establishments studied revealed 11.02 % organic waste, 34% paper and paper products, 18% plastics, 8.32% textiles, 6.6% construction debris, 0.8% pharmaceuticals, 0.4% electronics, and 12% other wastes (Fig. 6). The highest fraction of paper and paper products and plastics came from schools; plastics from shops and hospitals; pharmaceuticals from medical shops and hospitals and organic waste from hotels and restaurants. Compared to household and institutional waste, a high rate of glass in commercial waste was recorded. Institutional wastes included 45% paper and paper products, 22% organic waste, and 21% plastics. This study also found that 43% of commercial wastes were organic wastes, 23% were paper and paper products, and 22% were plastics. Hospital wastes, obsolete pesticides, and a few industrial wastes were considered to be the most common sources of hazardous waste in BM. It was reported that the waste generated by one of the biggest hospitals in Lamjung in BM was 141.765 kg/day, consisting of 91.94% nonhazardous waste and 8.060% hazardous waste [3]. In BM, most hospitals, health posts, and health clinics did not have incinerators. These wastes were not collected and disposed of separately, they were mixed with MSW. On average, MSW in 53 municipalities consisted of 56% organic waste, 16% plastic waste, and 16% paper and paper products [9].

Practices of SWM in urban areas

The municipality and private waste collectors are responsible for the collection of waste from urban areas of BM. The private waste collectors collect solid waste from the streets, collection centres, and dustbins in business and residential areas. After collecting the waste, it is transferred to a dumping site for disposal. Solid waste collection and transportation systems in urban areas of BM are presented in Fig. 7. WM employees are responsible for collecting and transporting solid waste from urban areas, where the roads are accessible by the schedule of the area. Thus, the waste is gathered from different collection points and finally transported to the dumping sites on the bank of the Marshyangdi River. However, waste generators are charged a waste collection fee. Even though there are rules and regulations governing the management of solid waste from the point of generation to disposal, some households, and commercial and

industrial solid waste generators were found to burn and bury their waste using unfriendly methods to control environmental pollution. MSW is not currently subject to recycling or recovery practices in BM.

In Fig. 8, it is evident that waste is being dumped in open areas without any engineering or scientific methodology. Almost all raw wastes from BM are dumped on the river banks, whereas villagers dump their household wastes near public places, spring banks or their land or burn. In urban areas, a tractor with a 2.5-m^3 container is employed in the transportation of solid waste for 25 tips per week by the private waste collector, authenticated by the municipality. There was an estimated 4,285 kg/day of solid waste generated in urban areas of BM. Interestingly, 62.218% of solid waste was generated in urban areas compared to village areas. In our study, it was not observed that the minimization, reuse, and recycling of municipal wastes. The results of this investigation demonstrated the municipal system for managing solid waste to be wholly ineffective. To stop the BM from getting further worse, immediate action must be taken to improvise. The municipality has also concurred with the statement and is aware that the existing practice is unhealthy and harmful to the environment. It can be suggested that the best method of disposing of solid waste is to compost it and use it as a soil amendment. As a result, less waste will need to be hauled to the landfill, prolonging its life [11]. When a large fraction of solid waste is organic, composting is one of the most sustainable methods of managing it [1]. A study by Timilsina [12] found that 21% of the waste from Kathmandu could be recycled and 62% of it could be composted. As an alternative to landfilling, aerobic composting is becoming increasingly important around the world [1] and is suggested in the BM.

Response to the current waste disposal system

Solid waste composition plays an important role in deciding which disposal method to adopt for a particular community from a technical perspective. Waste disposal activities in the municipality at present are not technically feasible or environmentally friendly. Questionnaires were given randomly to the heads of households in rural and urban areas regarding the waste disposal in BM, and the results obtained from this survey are listed in Fig. 9. The majority of the respondents (60%) disagreed with the view that the current dumping sites along Marsyangdi and other small river banks, springs, and public areas should not

be used for waste disposal. It was agreed by 48% of respondents that waste disposal along riversides impacts heritage, culture, religion, and the environment along with human health. Similarly, 50% of respondents agreed that waste should be separated before being disposed of. Only 15% of the respondents had no idea about waste treatment before disposal. There were approximately equal percentages of respondents who agreed or disagreed with the performance of private waste collectors. However, 33% of respondents agreed that BM should play a key role in WM practices. A large number of respondents to the questionnaire survey had no knowledge of sustainable WM systems. Based on the results of this study, it is clear that the municipality does not possess the necessary policies, finances, and technical capacities to establish and maintain a new landfill site.

Impact on the environment and public health

Waste treatment and final disposal systems in BM are primarily open dumping and burning, and the management of solid waste is made worse by unsustainable practices that increase environmental degradation and the spread of diseases. There is a direct correlation between the composition of waste and the methods of disposal [11]. As a result of the combustion and decay of solid wastes in open spaces, gaseous emissions, particulates, and volatiles are released into the atmosphere, and seepage leads to biological and chemical contamination of soil and groundwater [27, 28]. However, the methane generated during the combustion of organic waste contributes to an increase in global warming and climate change [29]. Furthermore, high organic contents in solid waste will promote the growth of microbial pathogens and cause infections and diseases in surrounding people as well [30]. Medical waste from hospitals, clinics, and other sources can be highly hazardous since it may contain infectious diseases or toxic chemicals. Landfill leachate from open dump sites contains high concentrations of fluoride, chloride, nitrogen, ammonium, toxic metals, organic carbons, biological oxygen demand (BOD), and chemical oxygen demand (COD) [31,32], as well as methane, carbon dioxide, carbon monoxide, nitrogen, hydrogen sulfide, and ammonia, which are some of the landfill gasses produced by traditional landfill practices [1]. Water sources can be contaminated by leachate, resulting in a negative impact on agricultural resources. The burning of solid

wastes and some microbes has been shown to cause several health problems such as skin, eye and nasal irritations, digestive, allergies, and psychological and respiratory problems [33]. Besides that, uncontrolled hazardous and electronic wastes have been linked to a variety of health problems, including chemical poisoning, low birth rates, cancer, nausea, vomiting, malformations, and neurological disorders [34]. It is believed that mixing healthcare products and biomedical waste with domestic waste and disposing of it at dumping sites without treatment increases the risk of Hepatitis B and HIV [28].

Based on the analysis of 20 water samples taken in Besisahar city and its surroundings, Aryal [35] reported that most of the samples exceeded the threshold standards for *Escherichia coli*, total coliforms, iron, manganese, and selenium, possibly due to improper SWM. In another study by Ono et al. [36], water samples collected from the Kathmandu valley's water supply system contained one or more bacterial species in 43 out of 57 samples, of which 51% contained E. coli in 22 out of 43, and they concluded that diarrhoea was caused by contaminated drinking water. It is currently common practice to landfill solid waste on the banks of rivers, streams, and open fields without considering their negative impact on water resources or the health of surrounding residents.

Economic benefit from solid waste

WM costs can be reduced by recycling the waste as a resource, thus decreasing the amount of waste that needs to be disposed of. Organic waste composting can also significantly reduce the costs and environmental impacts in households, in the community, and at the municipal level [11]. As well, organic waste separation increases the market value and recycling potential of inorganic waste such as paper and plastic.

The private waste collectors are not a company but got a bid from the municipality. They are obtaining money from every home and other organizations as management fees. There is no facility for precious waste segregation for direct-to-market and utilization processes. It was observed that this is a very weak side of the BM and waste collector. Recycling municipal waste and producing energy from organic waste should be prioritized by the municipality. This study revealed that the municipality will earn money

from the recyclable waste and will get energy like cooking gas from the waste, which can make up for the energy consumption.

Several efforts have been made to develop scientific environmental controls and build a progressive landfill in BM. It has been found that the operation of distant disposal sites has been associated with high transportation costs, making it impossible to maintain environmentally friendly waste disposal practices. Additionally, reuse and recycling not only provide livelihoods for those concerned but also significantly reduce the amount of waste at dumping sites. SWM must be sustainable by involving the private sector in WM, educating the public on environmental issues, and enforcing strict environmental laws [37].

Linkages with SDGs

Open dump sites pose a serious health threat to people living nearby waste collection centres and dumping sites, as well as releasing solid waste into water bodies, causing environmental contamination [38]. Target 3.2 aims to end preventable deaths of children under 5 years of age. The consumption of contaminated water due to the mismanagement of solid wastes can cause chronic and enteric diseases. In Nepal, enteric fever and diseases related to drinking water are the main culprits for the deaths of children under 5 years [39]. Target 3.9 refers to reducing the number of deaths and diseases due to hazardous chemicals, pollution, and contamination of air, water, and soil [13]. Aryal et al. [3] reported that medical waste disposal in BM is often problematic. It is important to properly manage medical waste within health facilities, such as by incineration, sterilization, or shredding, to reduce the spread of infections and pathogens. A lack of proper hazardous waste separation practices still poses a significant risk of illness and infection to garbage collectors in the study area. The fact that children and household members continue to suffer from chronic diseases indicates that SDG 3 has not been achieved.

Target 6.3 addresses enhancing water quality by reducing pollution, eliminating dumping, minimizing releases of chemicals and hazardous substances, substantially increasing recycling, and ensuring safe reuse [13]. It is recommended by Rodic and Wilson, [40] that all wastes, whether chemical or biological, be managed environmentally soundly, particularly those that end up in rivers, streams, or underground water. For instance, plastics do not degrade but turn into nanoparticles less than 2.5 mm in

size, which then enters the food chain. The consumption of drinking water that is contaminated with nano-plastics contributes to different cancers and hormonal disorders [42]. The majority of solid waste has been managed near water resources in our study area, making it almost impossible to achieve this goal in the near future. This study suggests that MSWs from BM contain a high level of organic waste, which will be turned into organic fertilizers as well as energy to achieve Target 6.3.

Target 13.3 refers to increased knowledge and capacity to deal with climate change [13]. MSW disposal facilities in developing countries are often open dumpsites, and the organic waste releases greenhouse gases, mainly methane, which contributes to air pollution and climate change. Rodic and Wilson, [40] mentioned that the disposal of solid waste in a well-controlled manner and the reduction of open burning can reduce climate change. A solution that can be suggested is to reduce waste to dumping sites and burn it as well as recycle corresponding wastes to achieve SDG 13, target 13.3.

Conclusions and future recommendations

The management of solid waste is still a major challenge for both local governments and private waste managers in developing economies. As a result of this work, it provides a clearer picture of WM by primarily collecting and separating waste at household and local levels. As determined by the waste composition study, household solid waste was composed of 68.03% organic, 8.13% paper, 5.5% agricultural, 5.21% plastic, 3.81% construction debris, 2.72% textile, 1.01% glass, 0.54% metal, and 0.1% rubber, 0.1% pharmaceutical, 0.05% electronic, and 4.8% other materials. A study conducted in BM revealed that household solid waste was generated at 197.604 g/capita/day. The waste composition in institutional and commercial establishments revealed 11.02% organic waste, 34% paper and paper products, 18% plastics, 8.32% textiles, 6.6% construction debris, 0.8% pharmaceuticals, 0.4% electronics, and 12% other wastes. Around 4.285 tons-solid waste/day were generated in urban areas, while 6.887 tons-solid waste/day were estimated for the whole municipality. The majority of the respondents (60%) disagreed with the view that the current dumping sites along Marsyangdi and other small river banks, springs, and public areas should not be used for waste disposal A sustainable resource recovery process can be achieved

by optimizing waste separation and reducing the need for waste sorting and cleanup. A better WM system will result in higher living standards and better health for many people with limited services, prevent hazardous materials from entering water resources, contribute significantly to the reduction of climate change, and help restore the environment. A WM regulation should be implemented by BM and its authorities, and a community awareness program would make the community responsible for managing the waste as well. As a result of the study, the following recommendations are made for improving the current SWM system in BM.

1. Government agencies, institutions, and non-governmental organizations need to continuously educate the community about the effective handling of solid waste at the household level.

2. A change in the moral attitude of people should lead to the separation and segregation of waste at the location.

3. A sustainable approach to managing solid waste in BM is to compost organic waste and use it as organic fertilizer.

4. Increasing public awareness about recycling materials such as metals and plastics at the point of origin.

5. Private companies must be involved in SWM to achieve sustainability.

6. Municipalities should implement strong rules and regulations to prevent the illegal dumping of solid waste along riverbanks and in public places.

Acknowledgements

The authors are very grateful for the support from the BM, private waste collectors, and residents for the information they provided through interviews.

Data Availability Statement

The manuscript contains all relevant data.

Funding

No specific funding was received for this study.

Competing interests

It has been declared that the authors have no competing interests.

Authors' contributions

MA and SA collected data, conducted field surveys, analyzed the data, and drafted the manuscript. All authors reviewed and approved the final manuscript.

References

1. Pokhrel D, Viraraghavan T. Municipal solid waste management in Nepal: Practices and challenges. Waste Manag 2005; 25: 555–562.
2. Aich A, Ghosh SK. Organic fraction of municipal solid waste – a valuable source of green energy in India. Int J Energy Sect Manag 2016; 10 (4): 526-545.
3. Aryal M, Adhikary S, Aryal V, Adhikari R. An analysis of solid waste management practices in Lamjung District Community Hospital, Nepal. J Wastes Biomass Manag 2019; 1(2): 9-13.
4. Laor P, Suma Y, Keawdounglek V, Hongtong A, Apidechkul T, Pasukphun N. Knowledge, attitude and practice of municipal solid waste management among highland residents in Northern Thailand. J Heal Res 2018; 32(2): 123-131.
5. Nnaji CC. Status of municipal solid waste generation and disposal in Nigeria. Manag Environ Qual 2015; 26(1): 53-71.
6. Alhumoud JM, Al- Kandari FA. Analysis and overview of industrial solid waste management in Kuwait. Manag Environ Qual 2008; 19 (5): 520-532.
7. Tinmaz E, Demir I. Research on solid waste management system: To improve existing situation in Corlutown of Turkey. Waste Manag 2006; 26: 307-314.
8. Tsai T. The impact of social capital on regional waste recycling. Sustain Devt 2008; 16 (1): 44-55.
9. ADB. *Asian Development Bank. Solid waste management in Nepal: Current status and policy recommendations*. Mandaluyong City, Philippines 2013; 1-69.
10. UNEP. *Nepal: State of the environment 2001*. Published by United Nations Environment Programme (UNEP) in colla, 2001.

11. Dangi MB, Urynowicz MA, Belbase S. Characterization, generation, and management of household solid waste in Tulsipur, Nepal. Habitat Int 2013; 40: 65-72.
12. Timilsina BP. Public and private sector involvement in municipal solid waste management: An overview of strategy, policy and practices. A Publication of Ministry of Population and Environment, Nepal. A Journal of the Environment 2001; 6 (7): 68–77.
13. UN. United Nation. Sustainable Development Goals. www.un.org/sustainabledevelopment/ sustainable-development-goals/ (accessed on 1 December 2016).
14. Ferronato N, Torretta V. Waste mismanagement in developing countries: A review of global issues. Int J Environ Res Public Heal 2019; 16(6): 1060.
15. Wilson DC, Velis CA. Waste management-Still a global challenge in the 21st century: An evidence-based call for action. Waste Manag Res 2015; 33 (12): 1049–1051.
16. NPC. National planning commission: Nepal's sustainable development goals, baseline report. Government of Nepal, National Planning Commission, Kathmandu, Nepal; 2017.
17. BMP. Besisahar municipality profile. January 2021. https://besishaharmun.gov.np.
18. NPHC. National Population and Housing Census. National statistics office, Government of Nepal. 2021. https://censusnepal.cbs.gov.np.
19. Zurbruegg C. *Solid waste management in developing countries: A sourcebook for policymakers and practitioners: EAWAG/SANDEC*; 2003.
20. Pathak DR, Mainali B, El Naga HA, Angove M, Kong I. Quantification and characterization of the municipal solid waste for sustainable waste management in newly formed municipalities of Nepal. Waste Manag Res 2020; 1–12.
21. Dahal Y, Adhikari B. Characterization and Quantification of Municipal Solid Waste in Jeetpur Simara Sub- Metropolitan City, Nepal. Hydro Nepal: J Water Energy Environ 2018; 22: 45–47.
22. Dangi MB, Pretz CR, Urynowicz MA, Gerow KG, Reddy JM. Municipal solid waste generation in Kathmandu, Nepal. J Environ Manag 2011; 92: 240-249.

23. Bolaane B, Ali M. *Sampling household waste at source: Lessons learnt in Gaborone.* Waste Manag Res 2004; 22(3): 142–148.

24. Tchobanoglous G, Theisen H, Vigil S. *Integrated solid waste management: Engineering principles and management issues*. New York, NY: McGraw-Hill, 1993.

25. Han X, Hu C, Lin L. A study on the impact of China’s urbanization on the quantity of municipal solid waste produced. Waste Manag Res 2020; 38: 184–192.

26. NEC. National economic census 2018. National report 1-1. Central Bureau of Statistics. Government of Nepal. August 24, 2022.

27. Khalil N, Khan M. A case of a municipal solid waste management system for a medium-sized Indian city, Aligarh. Manag Environ Qual 2009; 20(2): 121-141.

28. Pujara Y, Pathak P, Sharma A, Govani J. Review on Indian municipal solid waste management practices for reduction of environmental impacts to achieve sustainable development goals. J Environ Manag 2019; 248: 109238.

29. Çetinkaya AY, Bilgili L, Kuzu SL. Life cycle assessment and greenhouse gas emission evaluation from Aksaray solid waste disposal facility. Air Qual Atmos Heal 2018; 11: 549–558.

30. Fan YV, Lee CT, Klemes JJ, Chua LS, Sarmidi MR, Leow CW. Evaluation of effective microorganisms on home scale organic waste composting. J Environ Manag 2018; 216: 41–48.

31. Karak T, Bhattacharyya P, Das T, Paul RK, Bezbaruah R. Non-segregated municipal solid waste in an open dumping ground: A potential contaminant in relation to environmental health. Int J Environ Sci Technol 2013; 10(3):1-16.

32. Torretta V, Ferronato N, Katsoyiannis IA, Tolkou AK, Airoldi M. Novel and conventional technologies for landfill leachates treatment: A review. Sustainability 2017; 9 (1): 9.

33. Salemdeeb R, Ermgassen EKHJ, Kim MH, Balmford A, Al-Tabbaa A. Environmental and health impacts of using food waste as animal feed: A comparative analysis of food waste management options. J Clean Prod 201; 140: 871–880.

34. Pathak P, Srivastava R, Ojasvi R. Assessment of legislation and practices for the sustainable management of waste electrical and electronic equipment in India. Renew Sustain Energ Rev 2017; 78: 220–232.

35. Aryal M. An analysis of drinking water quality parameters to achieve sustainable development goals in rural and urban areas of Besisahar, Lamjung. Nepal. World Water Policy 2022; 1–27.

36. Ono K, Rai SK, Chikahira M, Fujimoto T, Shibata H, Wada Y, Tsuji H, Oda Y, Rai G, Matsumara T, Hotta H, Kawamura T, Uga S. Seasonal distribution of enteropathogens detected from diarrheal stool and water samples collected in Kathmandu, Nepal. SE Asian J Trop Med Publ Heal 2001; 32 (3): 520–526.

37. Thapa GB. Lessons learned from solid waste management in Kathmandu, Nepal. Habitat Int 1998; 22 (2): 97–114.

38. Gutberlet J, Baeder AM. Informal recycling and occupational health in Santo André, Brazil. Int J Environ Heal Res 2008; 18 (1): 1-15.

39. Adhikari D, Aryal M. A study of enteric fever and antimicrobial susceptibility patterns at the Manmohan Memorial Community Hospital in Kathmandu, Nepal. J Healthcare Develop Countries 2022; 1(2): 17-20.

40. Rodi´c L, Wilson DC. Resolving governance issues to achieve priority sustainable development goals related to solid waste management in developing countries. Sustainability 2017; 9: 404.

41. Lamizana B. Plastic planet: How tiny plastic particles are polluting our soil. *UN environment program, ecosystems and biodiversity*, 2013.

42. Elsheekh KM, Kamel RR, Elsherif DM, Shalaby AM. Achieving sustainable development goals from the perspective of solid waste management plans. J Eng Appl Sci 2021; 68: 9.

Figure legends

Fig. 1. (a) Location of BM in Nepal, and (b) map of BM.

Fig. 2. Collection of solid wastes in urban areas.

Fig. 3. Waste composition generated in BM.

Fig. 4. Average per capita household waste generation.

Fig. 5. Waste generation by household with average monthly expenditure.

Fig. 6. Composition of institutional, commercial and industrial wastes in BM.

Fig. 7. Flow chart of typical solid waste collection and transportation systems in urban areas.

Fig. 8. Solid wastes dumped and disposed of in various locations around BM.

Fig. 9. A response regarding waste disposal in BM.

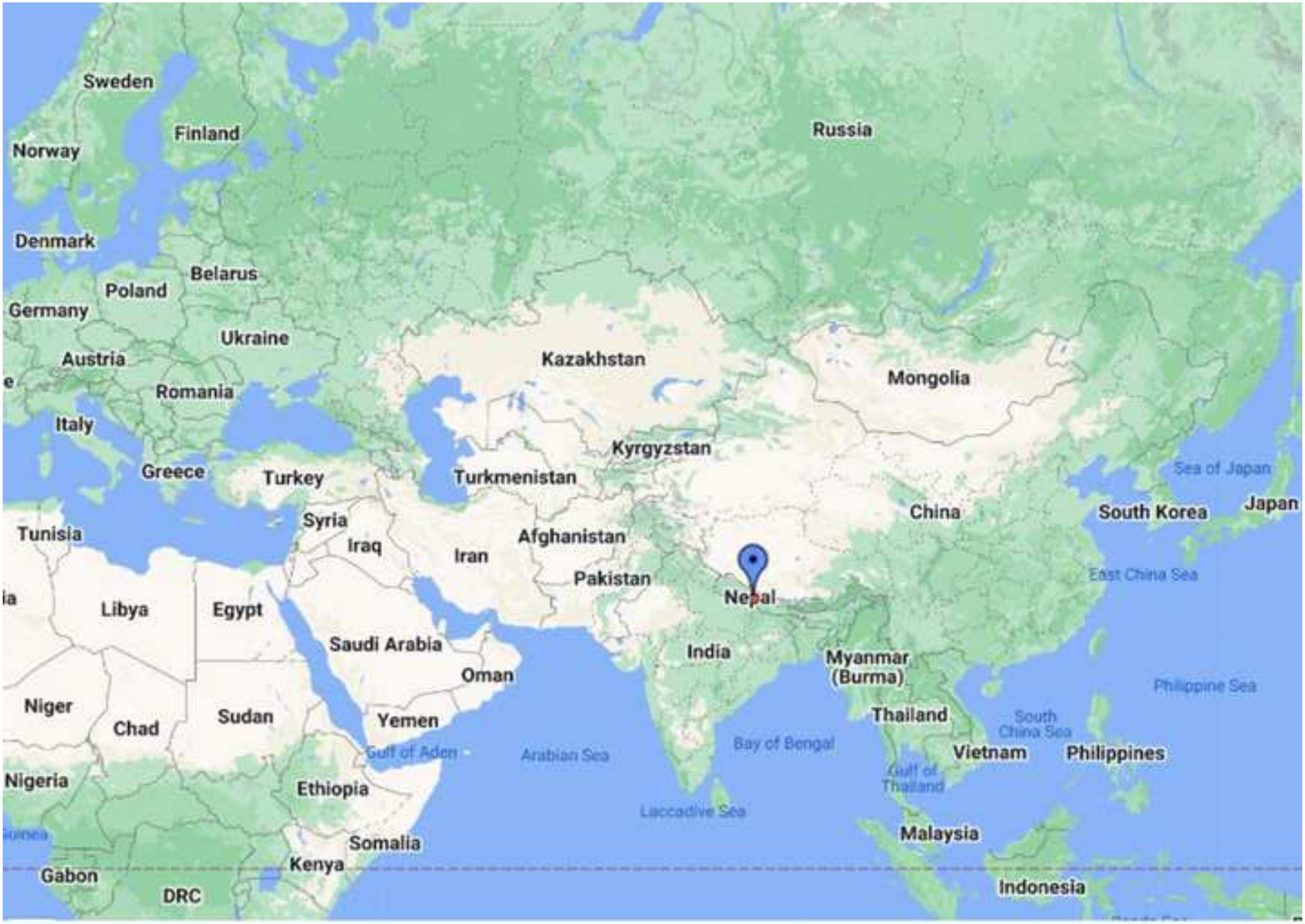
Sweden
Finland
Norway
Russia
Denmark
Belarus
Poland
Germany
Ukraine
Austria
Kazakhstan
Mongolia
Romania
Italy
Kyrgyzstan
Greece
Turkey
Turkmenistan
Sea of Japan
South Korea
Japan
China
Tunisia
Syria
Iraq
Afghanistan
Iran
Pakistan
East China Sea
Nepal
Libya
Egypt
Saudi Arabia
India
Myanmar (Burma)
Oman
Philippine Sea
Niger
Chad
Sudan
Yemen
Thailand
South China Sea
Gulf of Aden
Arabian Sea
Bay of Bengal
Vietnam
Philippines
Nigeria
Ethiopia
Gulf of Thailand
Laccadive Sea
Malaysia
Somalia
Kenya
Gabon
DRC
Indonesia

Fig. 1 (a)

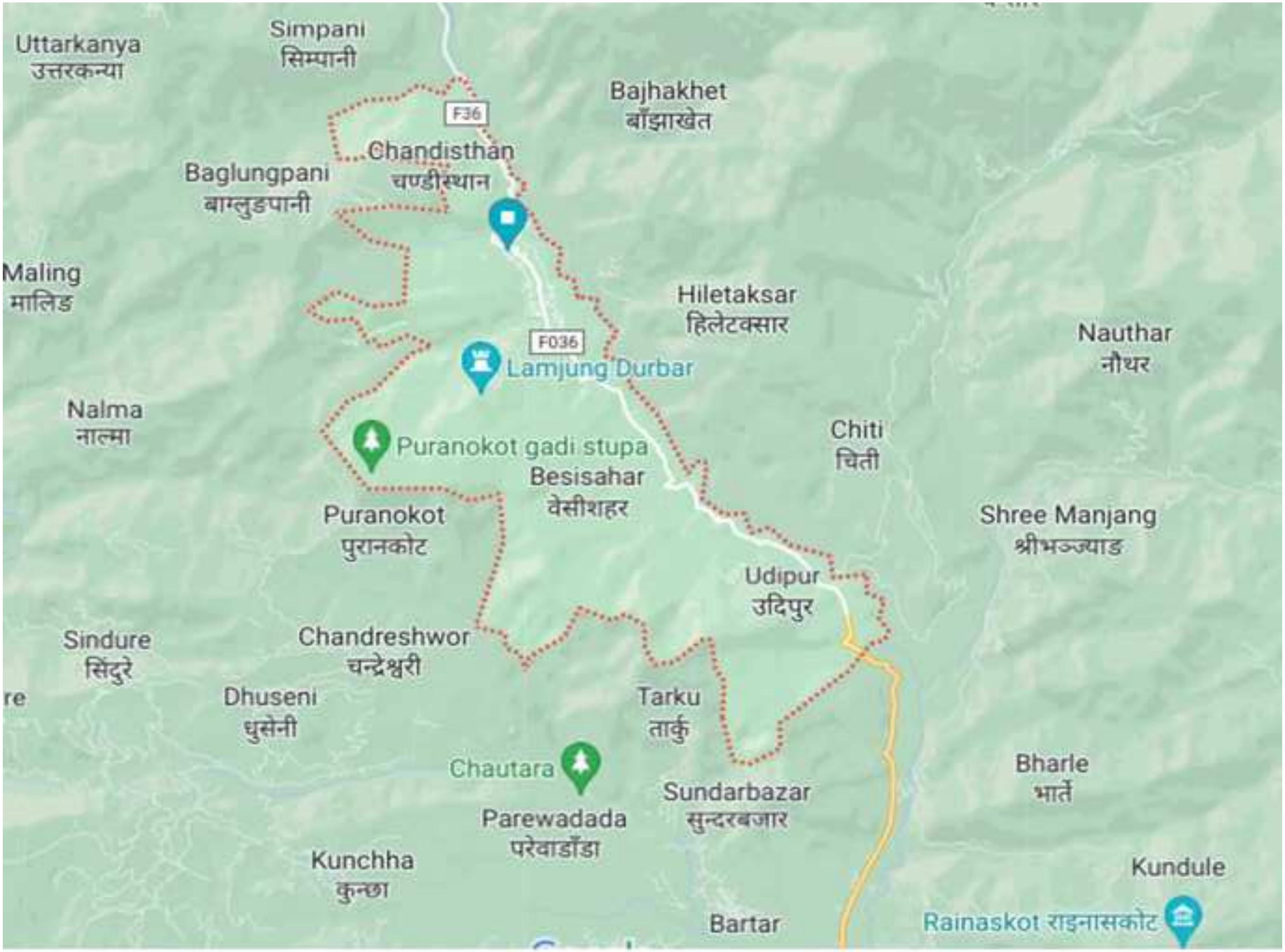
Uttarkanya
उत्तरकन्या
Simpani
सिम्पानी
F36
Bajhakhet
बाँझाखेत
Chandisthan
चण्डीस्थान
Baglungpani
बाग्लुङपानी
Maling
मालिङ
Hiletaksar
हिलेटक्सार
F036
Lamjung Durbar
Nauthar
नौथर
Nalma
नाल्मा
Chiti
चिती
Puranokot gadi stupa
Besisahar
वेसीशहर
Puranokot
पुरानकोट
Shree Manjang
श्रीमञ्ज्याङ
Udipur
उदिपुर
Sindure
सिंदुरे
Chandreshwor
चन्द्रेश्वरी
Dhuseni
धुसेनी
Tarku
तार्कु
Chautara
Bharle
भार्ले
Sundarbazar
सुन्दरबजार
Parewadada
परेवाडाँडा
Kunchha
कुन्छा
Kundule
Bartar
Rainaskot राइनासकोट

Fig. 1 (b)

Figure 2

Click here to access/download;Figure;Fig. 2.tif

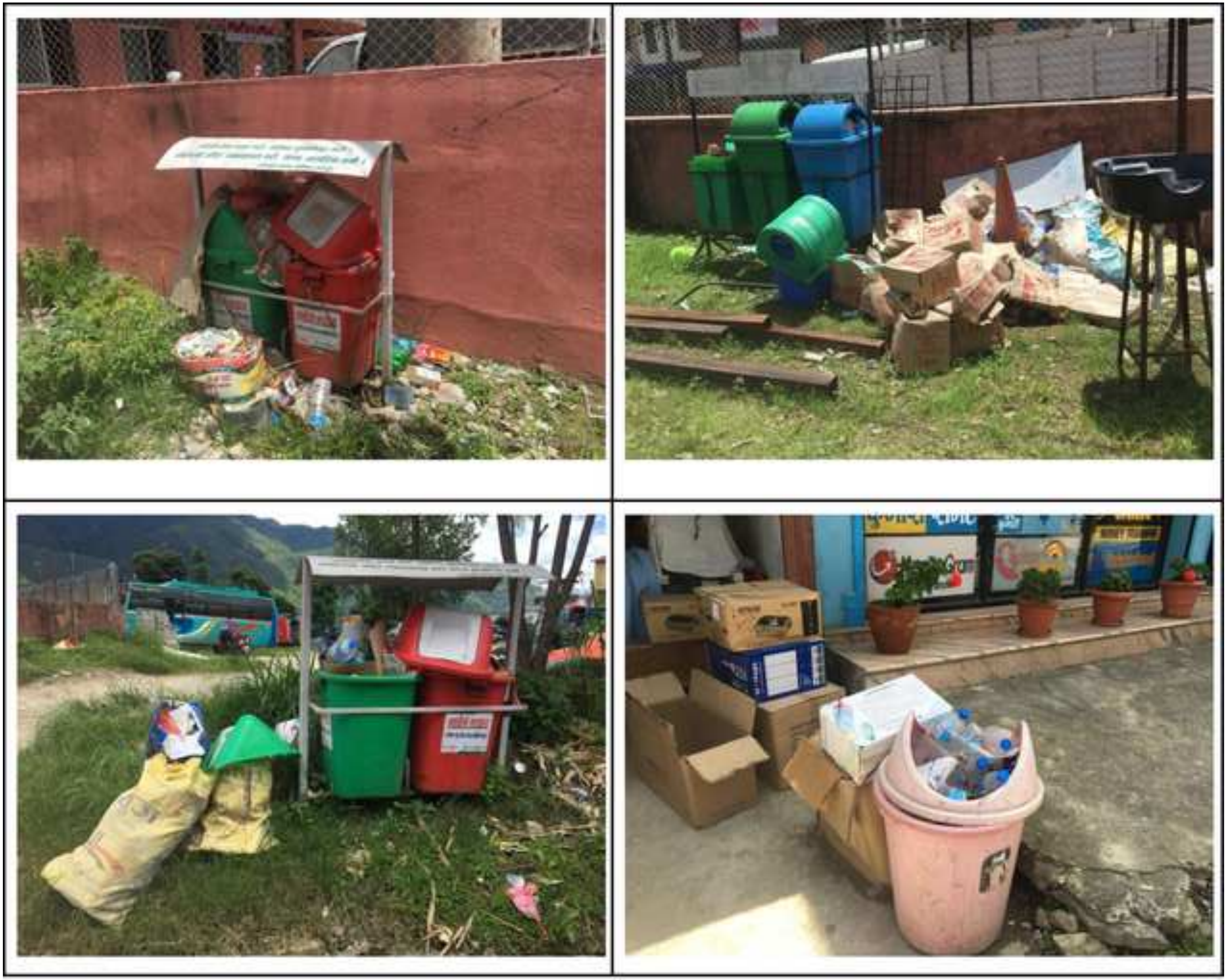

Fig. 2

 Click here to access/download;Figure;Fig. 3.tif

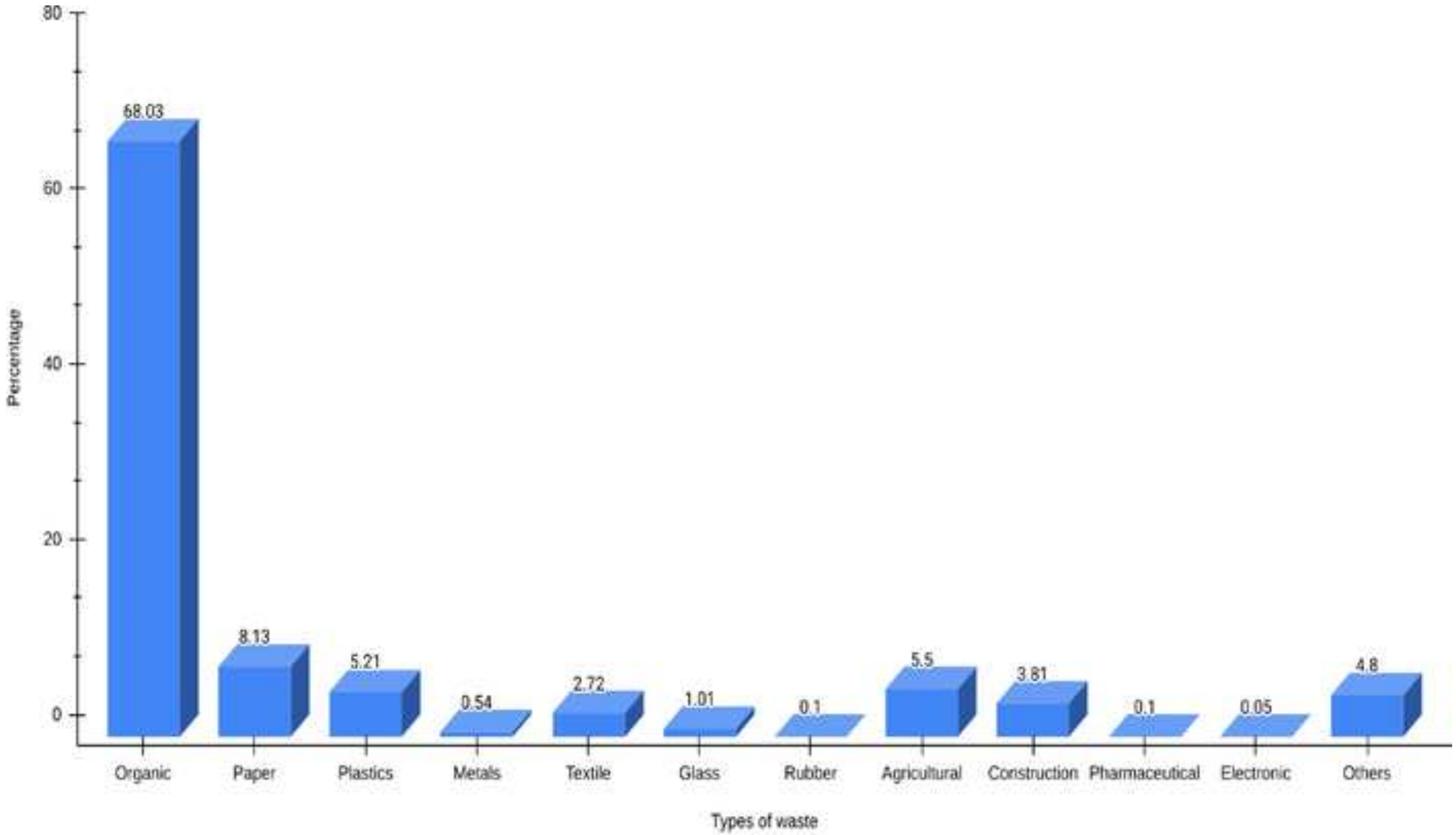

Fig. 3.

Figure 4

Click here to access/download;Figure;Fig. 4.tif

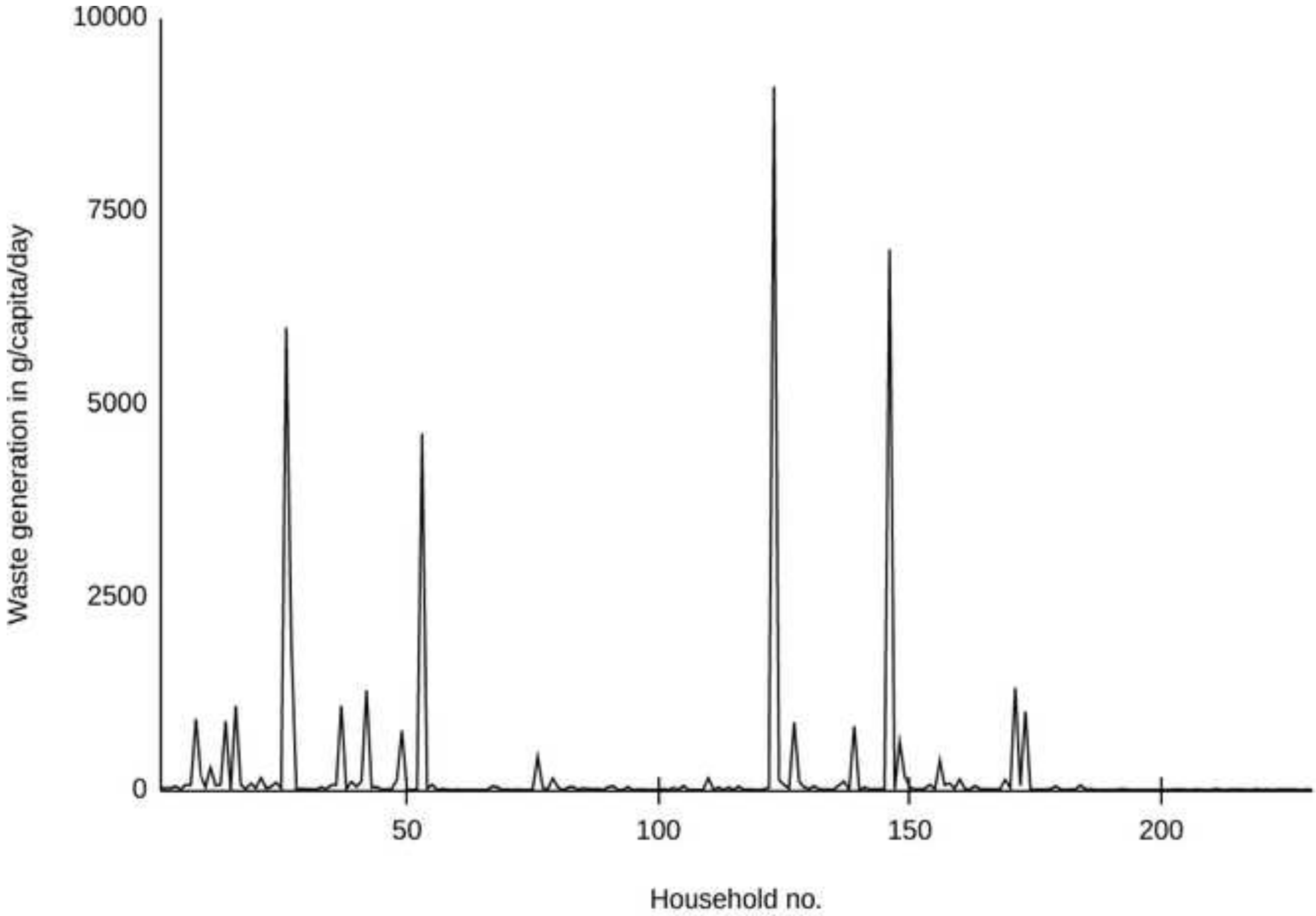

Fig. 4.

Click here to access/download;Figure;Fig. 5.tif

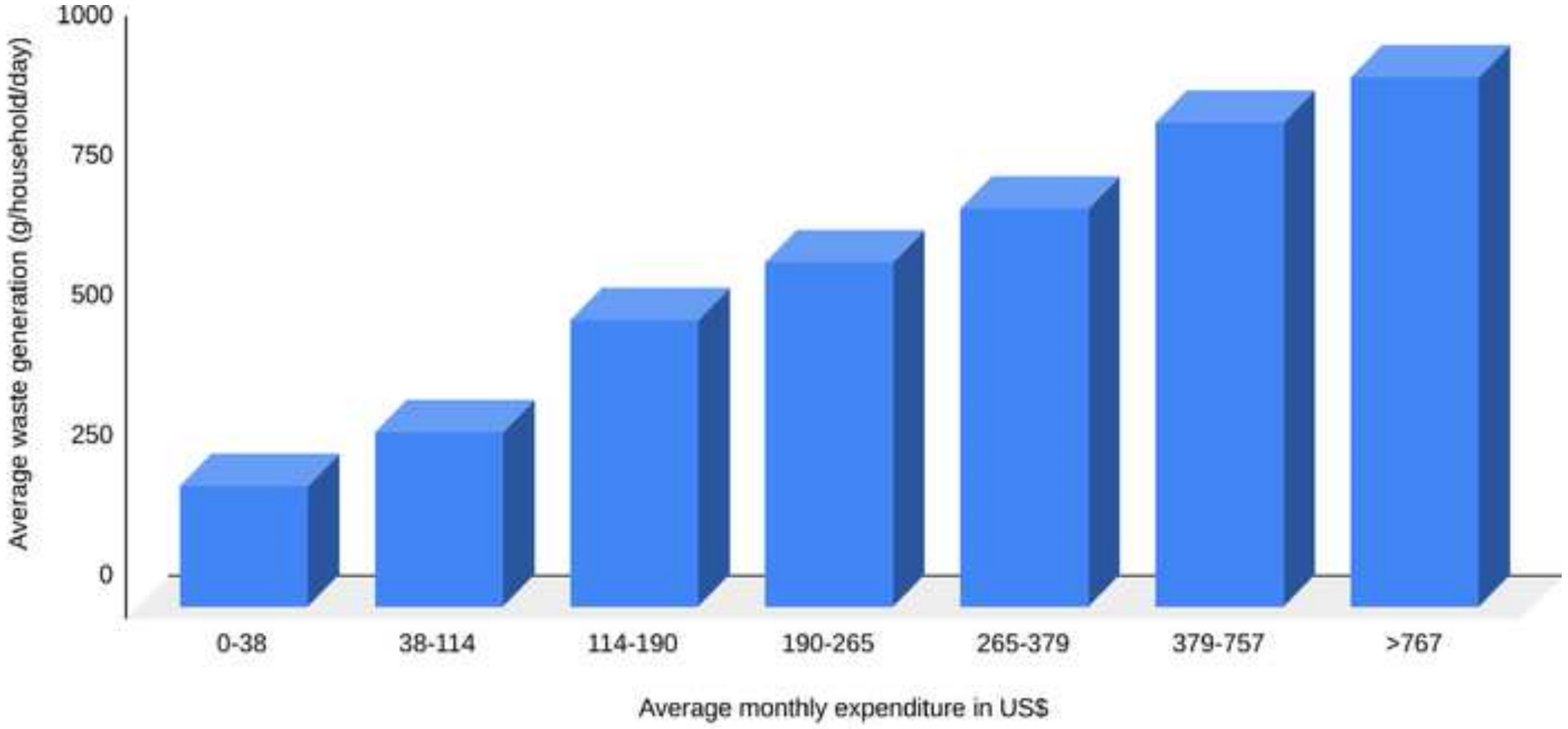

Fig. 5.

Figure 6

Click here to access/download;Figure;Fig. 6.tif

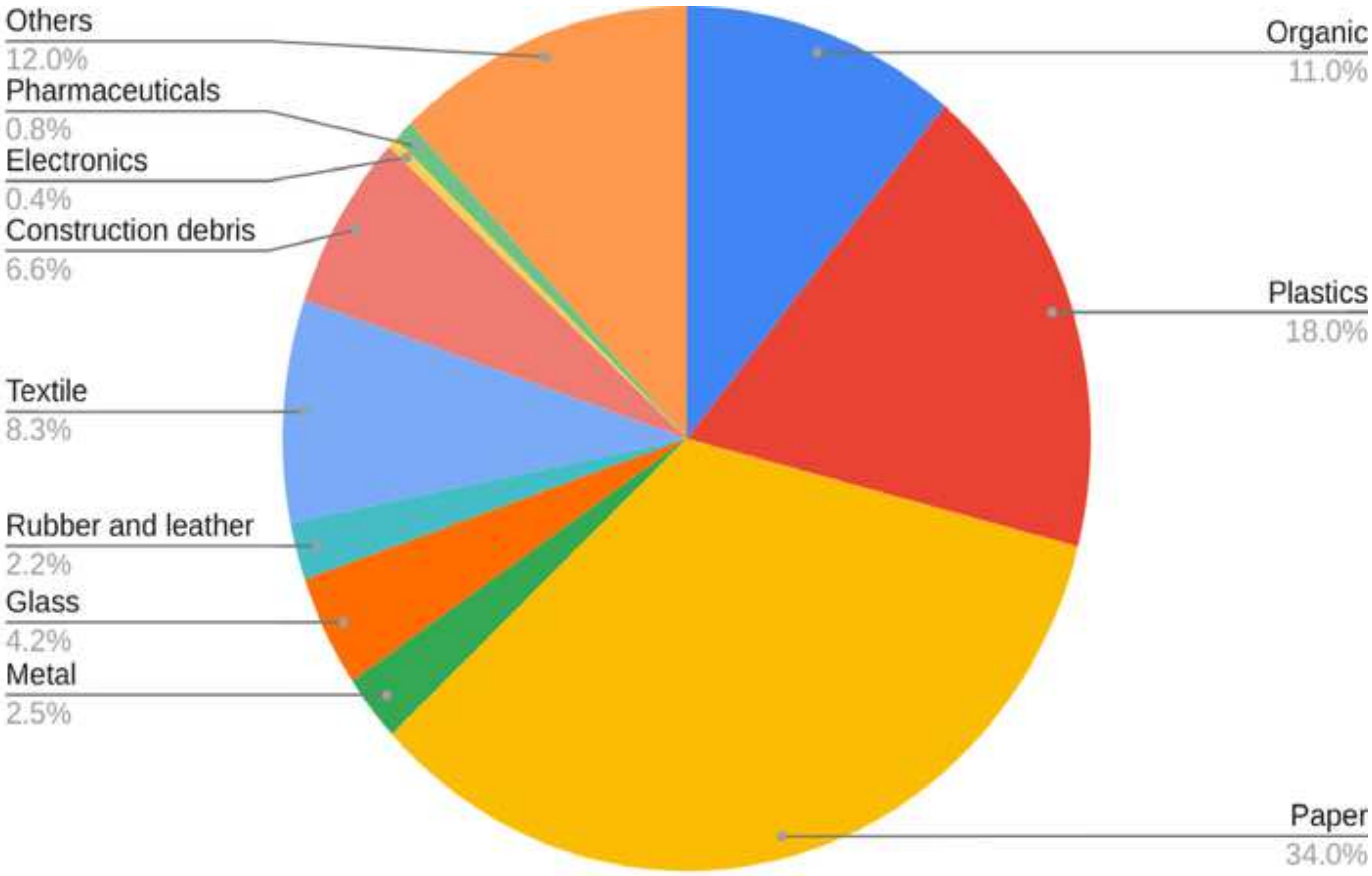

Fig. 6.

Click here to access/download;Figure;Fig. 7.tif

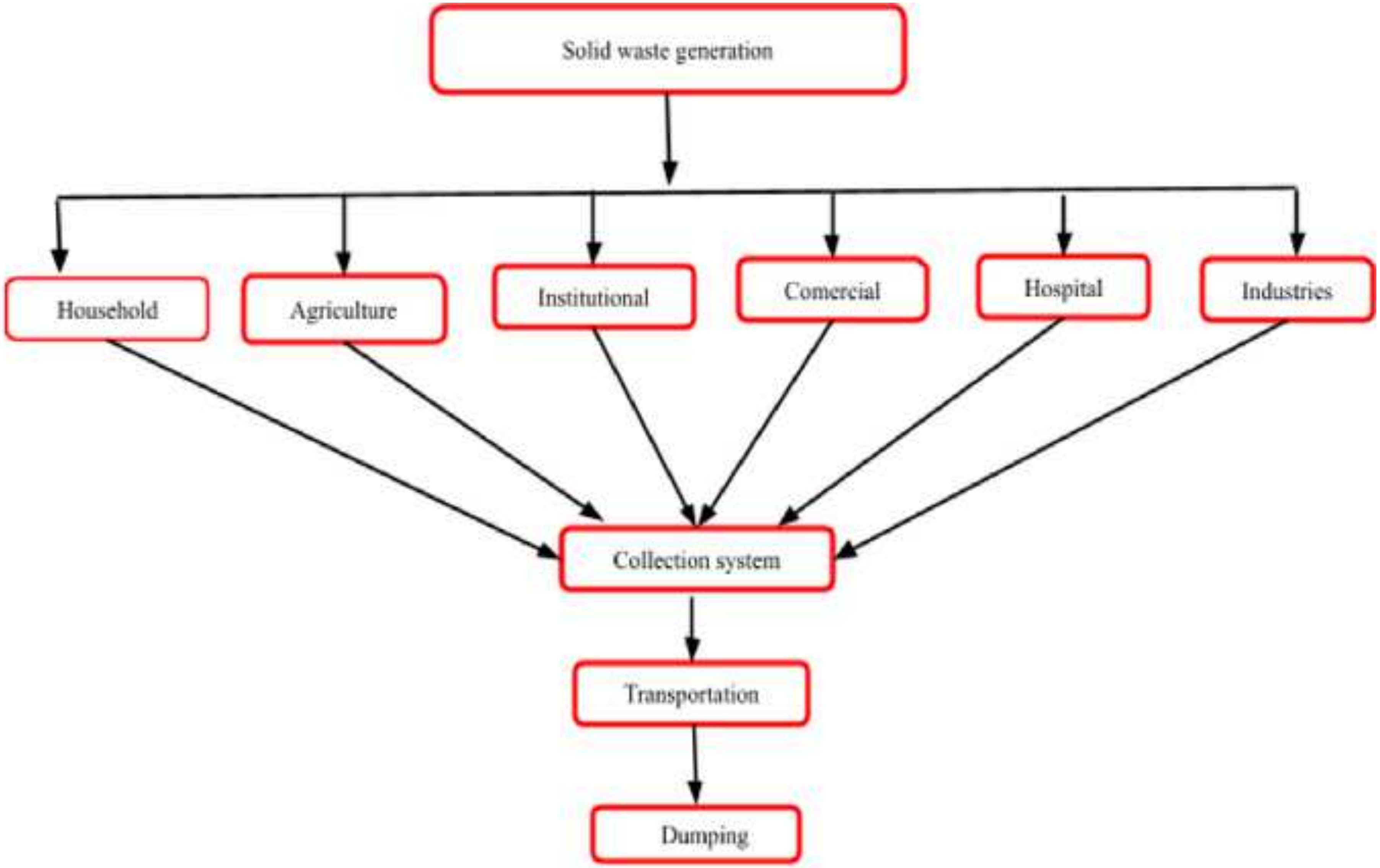

Fig. 7.

Click here to access/download;Figure;Fig. 8.tif

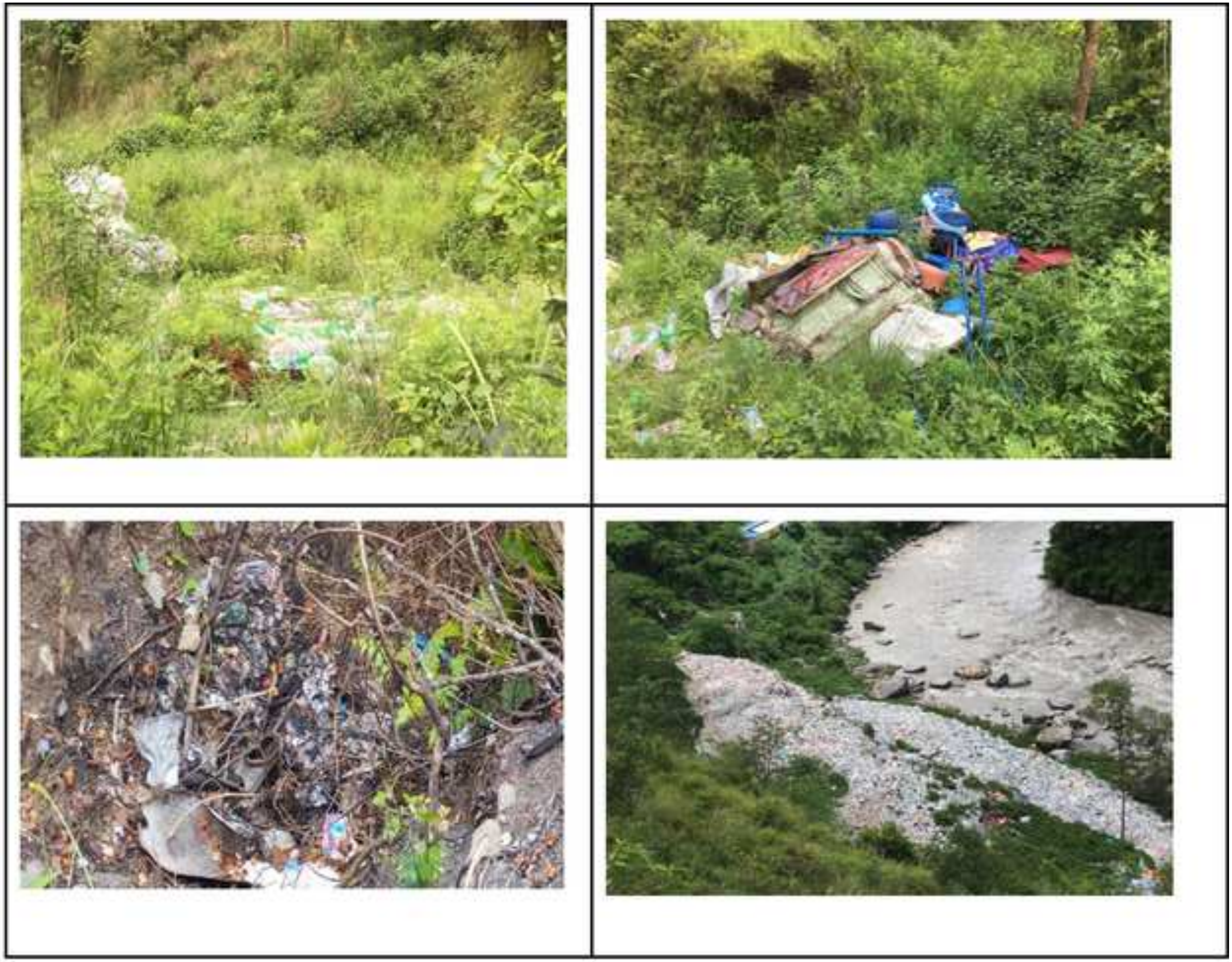

Fig. 8.

Figure 9

Click here to access/download;Figure;Fig. 9.tif

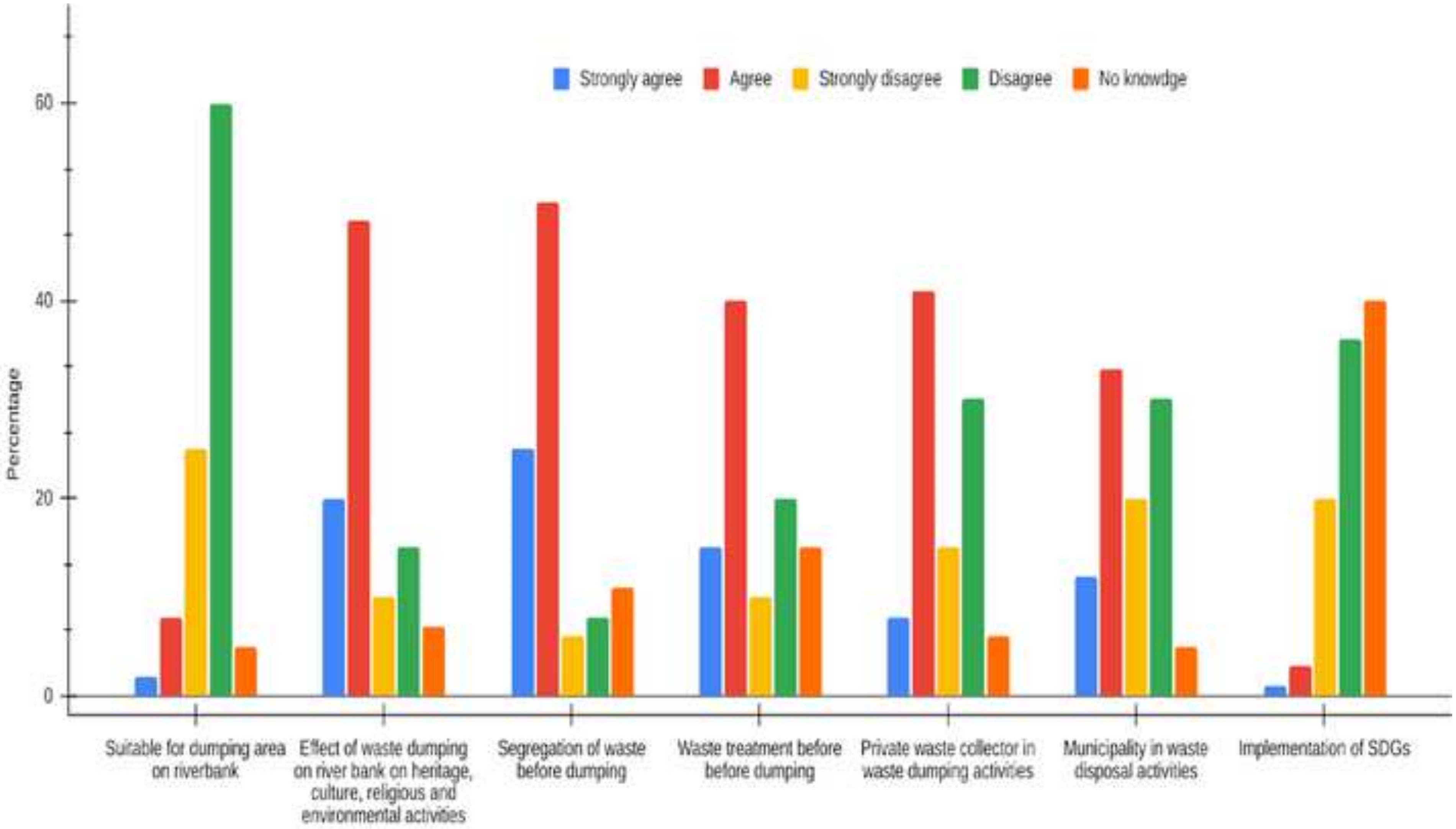

Fig. 9.
