# Supplementary material for: Solid waste management practices and challenges in Besisahar municipality, Nepal
Source: PLoS One. 2024 Mar 21;19(3):e0292758. doi: 10.1371/journal.pone.0292758 (PMC10956786; doi:10.1371/journal.pone.0292758)
Supplement: Attachment — Submitted filename: Answer to reviewers comments.docx [file pone.0292758.s003.docx]

**Answer to reviewers' comments**

**Comments to manuscript No.:** PONE-D-23-04289

**Title**: Solid waste management practices and challenges in Besisahar municipality, Nepal.

**Editor Comments:**

Thank you for submitting your manuscript to PLOS ONE. After careful consideration, we feel that it has merit but does not fully meet PLOS ONE’s publication criteria as it currently stands. Therefore, we invite you to submit a revised version of the manuscript that addresses the points raised during the review process.

**Response:** We have made all the changes addressed by the reviewers and we hope that the revised manuscript is now suitable for publication in your journal.

***Journal Requirements:***

1. Please ensure that your manuscript meets PLOS ONE's style requirements, including those for file naming. The PLOS ONE style templates can be found at

https://journals.plos.org/plosone/s/file?id=wjVg/PLOSOne_formatting_sample_main_body.pdf and
<https://journals.plos.org/plosone/s/file?id=ba62/PLOSOne_formatting_sample_title_authors_affiliations.pdf>

**Response:** We have checked all according to the PLOS ONE style template.

2. You indicated that ethical approval was not necessary for your study. We understand that the framework for ethical oversight requirements for studies of this type may differ depending on the setting and we would appreciate some further clarification regarding your research. Could you please provide further details on why your study is exempt from the need for approval and confirmation from your institutional review board or research ethics committee (e.g., in the form of a letter or email correspondence) that ethics review was not necessary for this study? Please include a copy of the correspondence as an ""Other"" file.

**Response:** We have not mentioned this ethical approval in the manuscript. If any in the editorial manager then we will correct this.

3. In your Data Availability statement, you have not specified where the minimal data set underlying the results described in your manuscript can be found. PLOS defines a study's minimal data set as the underlying data used to reach the conclusions drawn in the manuscript and any additional data required to replicate the reported study findings in their entirety. All PLOS journals require that the minimal data set be made fully available. For more information about our data policy, please see <http://journals.plos.org/plosone/s/data-availability>.

4. We note that Figure 1a and 1b in your submission contain map/satellite images which may be copyrighted. All PLOS content is published under the Creative Commons Attribution License (CC BY 4.0), which means that the manuscript, images, and Supporting Information files will be freely available online, and any third party is permitted to access, download, copy, distribute, and use these materials in any way, even commercially, with proper attribution. For these reasons, we cannot publish previously copyrighted maps or satellite images created using proprietary data, such as Google software (Google Maps, Street View, and Earth). For more information, see our copyright guidelines: http://journals.plos.org/plosone/s/licenses-and-copyright.

We require you to either (1) present written permission from the copyright holder to publish these figures specifically under the CC BY 4.0 license, or (2) remove the figures from your submission:

(1) You may seek permission from the original copyright holder of Figure 1a and 1b to publish the content specifically under the CC BY 4.0 license.

We recommend that you contact the original copyright holder with the Content Permission Form (http://journals.plos.org/plosone/s/file?id=7c09/content-permission-form.pdf) and the following text:
“I request permission for the open-access journal PLOS ONE to publish XXX under the Creative Commons Attribution License (CCAL) CC BY 4.0 (http://creativecommons.org/licenses/by/4.0/). Please be aware that this license allows unrestricted use and distribution, even commercially, by third parties. Please reply and provide explicit written permission to publish XXX under a CC BY license and complete the attached form.”

Please upload the completed Content Permission Form or other proof of granted permissions as an ""Other"" file with your submission.

In the figure caption of the copyrighted figure, please include the following text: “Reprinted from [ref] under a CC BY license, with permission from [name of publisher], original copyright [original copyright year].”

(2) If you are unable to obtain permission from the original copyright holder to publish these figures under the CC BY 4.0 license or if the copyright holder’s requirements are incompatible with the CC BY 4.0 license, please either i) remove the figure or ii) supply a replacement figure that complies with the CC BY 4.0 license. Please check copyright information on all replacement figures and update the figure caption with source information. If applicable, please specify in the figure caption text when a figure is similar but not identical to the original image and is therefore for illustrative purposes only.

The following resources for replacing copyrighted map figures may be helpful:

USGS National Map Viewer (public domain): http://viewer.nationalmap.gov/viewer/
The Gateway to Astronaut Photography of Earth (public domain): http://eol.jsc.nasa.gov/sseop/clickmap/
Maps at the CIA (public domain): https://www.cia.gov/library/publications/the-world-factbook/index.html and https://www.cia.gov/library/publications/cia-maps-publications/index.html
NASA Earth Observatory (public domain): http://earthobservatory.nasa.gov/
Landsat: http://landsat.visibleearth.nasa.gov/
USGS EROS (Earth Resources Observatory and Science (EROS) Center) (public domain): http://eros.usgs.gov/#
Natural Earth (public domain): http://www.naturalearthdata.com/

**Response:** We have removed the Fig. 1 (a) and (b) from the manuscript since the given figures are the map of Nepal and Besisahar municipality and the readers can easily get them through a Google search.

**Reviewers' comments:**

**Reviewer #1:**

**General comments:**

The research is of great importance and contributes to waste management in Nepal, and works as a reference for policymakers in countries or areas that share similarities in municipal solid waste generation or management. However, the way how the authors display their research is limited to descriptive information, which lacks critical analysis, and key findings are not clearly presented.

**Response:** Thank you very much for your suggestion. We have made the revised manuscript according to your comments.

**Specific comments:**

1. **Please note that all tables are missing in the manuscript.**

**Response:** We have replaced some figures and added some tables in the appropriate places in the manuscript. We checked all the tables in the manuscript and could not find the errors in this regard.

We are thankful for the reviewers’ valuable comments.

2. **It would be helpful if line numbers could be provided.**

**Response:** We have inserted the line numbers in the revised manuscript. We apologise for missing this in the initial submission and pardon for this mistake.

Thank you very much for pointing out this mistake.

3, **The last paragraph of the introduction should state the purpose of this paper concisely.**

**Response:** We have added the following text in the last paragraph of the introduction part;

“This study focuses on examining the unique context of SWM in Besisahar municipality. It investigates WM techniques, infrastructure, resources, policies, and challenges specific to the area. The study evaluates present WM systems, including collection, transportation, and disposal. It identifies challenges such as inadequate garbage collection systems, a lack of public awareness, and financial limitations. In addition, it also provides valuable insights for policymakers, WM practitioners, and researchers by addressing these challenges and proposing recommendations and solutions to improve SWM in Besisahar. The study aims to promote sustainable and efficient WM strategies for the benefit of the municipality's residents and the environment.”

Thanks for the reviewers’ important comments.”

4. P**age 4, BMP: please spell out when it first appears in the text.**

**Response:** We have corrected this in the manuscript. Please, see the attached manuscript.

We are thankful for the reviewers’ suggestions.

5. **Page 5, three decimal places are unnecessary for percentages in the text, and it is better to keep one decimal for percentage data.**

**Response:** We have corrected the decimal in percentage as one decimal system on page no. 5 along with through ot the manuscript.

5. **Page 5, Please specify the qualitative and quantitative methods in this study.**

**Response:** We have added the qualitative and quantitative methods in the topic “Data analysis” in the “Method and material” section as “The analysis of the data was carried out using both quantitative (waste generation rates, cost-benefit analysis, and statistical analysis) and qualitative (interviews, case studies, and field surveys) methods. In this study, a significance level of p < 0.05 was adopted as the threshold for determining statistical significance. Arithmetic mean, median, standard deviation, skewness and kurtosis were determined. A Pearson correlation analysis (r) was conducted to determine the strength of the relationship between parameters related to SWM.” Please, see the revised manuscript.

6. **Page 6, Please specify sources of data when you mention them in the text, for example,**

**The results of the questionnaire study revealed that 72% of household respondents knew about waste separation, but they did not apply it practically, whereas only 28 % of respondents indicated that they were utilizing such knowledge to separate waste properly at the household level.**

**Response:** The study involved conducting surveys using questionnaires to gather information from household respondents. The questionnaire likely included questions related to knowledge and practices of waste separation at the household level. The specific sources of data used in the study may vary, and additional methods such as focus group discussions or literature reviews might have been employed to gather a comprehensive understanding of the solid waste management practices and challenges in Besisahar municipality.

We have restructured the text in Results and Discussion “Waste generation and segregation” as ”The findings of the questionnaire study indicated that a considerable portion of households, approximately 72%, possessed knowledge about waste separation. However, despite being aware of this concept, they did not translate their knowledge into practical implementation. Conversely, only 28% of the respondents indicated actively applying their knowledge to properly separate waste at the household level. “

We have also refreshed and added the following text in the last two paragraphs on the same topics as “Several waste types have been studied in some studies, encompassing organic waste, plastics, paper and paper products, metals, glass, rubber and leather, textiles, dirt and construction debris, hazardous wastes, and other forms of waste [9,21**]**. Notably, the classification of municipal solid wastes was expanded by Dangi et al. [11] and Dangi et al. [23] to include two new waste types, namely hazardous wastes, and dirt and building debris.

By categorizing their solid waste into several categories for collection and treatment, and encouraging the 3Rs (reduce, reuse, recycle), households can significantly improve waste management. A three-bin system, which allows residents to divide waste into dry recyclables, moist organic and/or domestic waste, and hazardous or residual waste, has been implemented in several places [9]. In Nepal, initiatives to encourage trash segregation have been gaining momentum. However, insufficient infrastructure, a lack of understanding, and restricted access to waste management services are frequently blamed for poor segregation practices. To improve waste segregation practices, it is essential to raise public awareness of its advantages, educate people about it, encourage public engagement, build segregation facilities, and develop the waste management system.”

Please, see the revised manuscript.

7. **It was reported that solid waste collection was implemented in wards numbers 1, 2, 3, 7, 8 and 9.**

**Response:** We have corrected it in Results and discussion in the section “Household SWM practices as “The field survey indicated that solid waste collection had been effectively implemented in wards 1, 2, 3, 7, 8, and 9, respectively.” We are very thankful for the reviewer’s suggestion.

**Reviewer #2:**

The following are the specific comments which need to be addressed before its publication;

1. **It’s very difficult to review the submitted manuscript without adding the line numbers, thus, I commented directly in the attached PDF through annotation tools. Authors must add the line number before submitting the revised version of the manuscript, if any.**

**Response:** We have added the line numbers in the revised manuscript. We are extremely sorry for this inconvenience.

2. **What are the differences between this study and others in the literature? The originality/novelty of the paper should be clearly stated in the introduction section.**
**Response:** We have added the following text in the last paragraph of the introduction part. Please, see the revised manuscript.

“This study focuses on examining the unique context of SWM in Besisahar municipality. It investigates WM techniques, infrastructure, resources, policies, and challenges specific to the area. The study evaluates present WM systems, including collection, transportation, and disposal. It identifies challenges such as inadequate garbage collection systems, a lack of public awareness, and financial limitations. In addition, it also provides valuable insights for policymakers, WM practitioners, and researchers by addressing these challenges and proposing recommendations and solutions to improve SWM in Besisahar. The study aims to promote sustainable and efficient WM strategies for the benefit of the municipality's residents and the environment. ”

We are thankful for the reviewer’s suggestion.
3. **Manuscript is completely lacking with citation of 2023. In addition, the authors have cited 2-3 references only for the year, 2022. The manuscript should cover the recent literature related to this subject. Introduction is completely lacking the citation from the years 2022; 2023.**

**Response:** We submitted this manuscript to the editorial manager on Feb 14 2023 and therefore we were unable to add the references from 2023. Now we have added the latest references to the introduction part. Please, see the reference section of the revised manuscript.

4. **Old references may be replaced with recent references.**

**Response:** We have removed the text “An investigation conducted in 1995 found that only 38% of the farmers buried or burned old or unused pesticides, whereas others were disposed of in waste containers as solid waste in open fields [10]” from the Results and discussion of topic “Households waste characterization”. Since this survey was conducted in 1995.

The following old references are replaced by the latest ones; Please, see the attached manuscript.

1. Aich A, Ghosh SK. Organic fraction of municipal solid waste – a valuable source of green energy in India. Int J Energy Sect Manag 2016; 10 (4): 526-545.

***Replaced by:***

Thabit Q, Nassour A, Nelles M. Facts and figures on aspects of waste management in Middle East and North Africa region. Waste 2023; 1(1), 52-80.

2. Laor P, Suma Y, Keawdounglek V, Hongtong A, Apidechkul T, Pasukphun N. Knowledge, attitude and practice of municipal solid waste management among highland residents in Northern Thailand. J Heal Res 2018; 32(2): 123-131.

***Replaced by:***

Khanal A, Giri S, Mainali P. The practices of at-source segregation of household solid waste by the youths in Nepal. J Environ Public Health. 2023; 5044295.

3. Nnaji CC. Status of municipal solid waste generation and disposal in Nigeria. Manag Environ Qual 2015; 26(1): 53-71.

***Replaced by:***

Ghulam ST, Abushammala H. Challenges and opportunities in the management of electronic waste and Its impact on human health and environment. Sustainability 2023; 15(3), 1837.

4. Alhumoud JM, Al‐ Kandari FA. Analysis and overview of industrial solid waste management in Kuwait. Manag Environ Qual 2008; 19 (5): 520-532.

***Replaced by:***

Wang Y, Shi Q. The impact of municipal solid waste sorting policy on air pollution: Evidence from Shanghai, China. PLoS ONE 2022; 17(11): e0277035.

5. Tinmaz E, Demir I. Research on solid waste management system: To improve existing situation in Corlutown of Turkey. Waste Manag 2006; 26: 307-314.

***Replaced by:***

Tangri N. Waste incinerators undermine clean energy goals. PLOS Clim 2022; 2(6): e0000100.

6. Tsai T. The impact of social capital on regional waste recycling. Sustain Devt 2008; 16 (1): 44-55.

***Replaced by:***

Xin C, Zhang T, Tsai S-B, Zhai Y-M, Wang J. An empirical study on greenhouse gas emission calculations under different municipal solid waste management strategies. Appl. Sci. 2020; 10(5), 1673.

7. UNEP. *Nepal: State of the environment 2001*. Published by United Nations Environment Programme (UNEP) in colla, 2001.

***Replaced by:***

CBS. 2020. Waste management baseline survey of Nepal 2020. Central Bureau of Statistics, Kathmandu, Nepal.

8. Wilson DC, Velis CA. Waste management-Still a global challenge in the 21st century: An evidence-based call for action. Waste Manag Res 2015; 33 (12): 1049–1051.

***Replaced by:***

Sharma HB, Vanapalli KR, Samal B, Cheela VRS, Dubey BK, Bhattacharya J. Circular economy approach in solid waste management system to achieve UN-SDGs: Solutions for post-COVID recovery. Sci Total Environ. 2021; 15: 800: 149605.

9. Zurbruegg C. Solid waste management in developing countries: A sourcebook for policymakers and practitioners: EAWAG/SANDEC; 2003.

***Replaced by:***

Eshete H, Desalegn A, Tigu F. Knowledge, attitudes and practices on household solid waste management and associated factors in Gelemso town, Ethiopia. PLoS ONE 2023; 18(2): e0278181.

10. Khalil N, Khan M. A case of a municipal solid waste management system for a medium-sized Indian city, Aligarh. Manag Environ Qual 2009; 20(2): 121-141.

***Replaced by:***

Chanana I, Sharma A, Kumar P, Lokender Kumar L, Kulshreshtha S, Kumar S. Patel SKS. Combustion and stubble burning: A major concern for the environment and human health. Fire 2023; 6(2): 79.

11. Salemdeeb R, Ermgassen EKHJ, Kim MH, Balmford A, Al-Tabbaa A. Environmental and health impacts of using food waste as animal feed: A comparative analysis of food waste management options. J Clean Prod 2017; 140: 871–880.

***Replaced by:***

Abubakar IR, Maniruzzaman KM, Dano UL, AlShihri FS, AlShammari MS, Ahmed SMS, Al-Gehlani WAG, Alrawaf TI. Environmental sustainability impacts of solid waste management practices in the global South. [Int J Environ Res Public Health.](https://www.ncbi.nlm.nih.gov/pmc/articles/PMC9566108/#) 2022; 19(19): 12717.

12. Gutberlet J, Baeder AM. Informal recycling and occupational health in Santo André, Brazil. Int J Environ Heal Res 2008; 18 (1): 1-15.

***Replaced by:***

Siddiqua A, Hahladakis JN, Al-Attiya WAKA. An overview of the environmental pollution and health effects associated with waste landfilling and open dumping. Environ Sci Pollut Res 2022; 29,: 58514–58536.

13. Lamizana B. Plastic planet: How tiny plastic particles are polluting our soil. UN environment program, ecosystems and biodiversity, 2013.

***Replaced by:***

Yuan Z, Nag R, Cummins E. Human health concerns regarding microplastics in the aquatic environment - From marine to food systems. Sci. Total Environ. 2022; 823: 153730.

14. Karak T, Bhattacharyya P, Das T, Paul RK, Bezbaruah R. Non-segregated municipal solid waste in an open dumping ground: A potential contaminant in relation to environmental health. Int J Environ Sci Technol 2013; 10(3):1-16.

***Replaced by:***

Makhatova A, Mazhit B, Sarbassov Y, Meiramkulova K, Inglezakis VJ, Poulopoulos SG. Effective photochemical treatment of a municipal solid waste landfill leachate. PLoS One. 2020 22;15(9):e0239433.

We are thankful for the reviewer’s valuable suggestion.

5. **The discussion needs improvement and should be linked to the findings of the previous reports on this topic. discussion is elaborative but it needs more adequate discussion with supporting latest references. Discussion should be according to the results.**

**Response:** We have added the following in the discussion part with the following references;

1. GoN, 2022. Solid Waste account for urban Municipalities of Nepal 2022. System of environMental econoMic accounting. Government of Nepal. National Planning Commission Central Bureau of Statistics Kathmandu, Nepal. CBS Publication.

2. Das, B., Bhave, P. V., Sapkota, A., Byanju, R.M., 2018. Estimating emissions from open burning of municipal solid waste in municipalities of Nepal. Waste Manag. 79, 481–490.

3. Hondo, D., Arthur, L., Gamaralalage, PJD. 2020. Solid Waste Management in Developing Asia: Prioritizing Waste Separation. Asian Development Bank Institute.<https://www.adb.org/sites/default/files/publication/652121/adbi-pb2020-7.pdf>.

We have added the elaborative discussion in the appropriate places in the revised manuscript. Please, see the attached manuscript.

**In the Abstract**

We have added, “An important correlation between the parameters of solid waste was found by statistical analysis.”

**In the introduction**

We have added as “In Nepal, the Solid Waste Management Act, 2011 AD, and the Solid Waste Management Rules, 2013 AD, serve as the governing regulations for the SWM sector. These regulations establish the guidelines and framework for managing solid waste in the country. According to these regulations, local governments are entrusted with the responsibility of collecting, transporting, and disposing of waste in an environmentally sustainable manner.”

**In statical analysis:**

We have inserted as “In this study, a significance level of p < 0.05 was adopted as the threshold for determining statistical significance. Arithmetic mean, median, standard deviation, skewness and kurtosis were determined. A Pearson correlation analysis (r) was conducted to determine the strength of the relationship between parameters related to SWM.”

**Results and discussion**

**Waste generation and segregation**

We have refreshed the text as” Waste segregation at the source is considered an important practice in solid waste management. However, studies have indicated low levels of waste segregation among households in Nepal [20]. The composition of solid waste generated in both rural and urban areas of BM was categorized and presented in Table 2.”

“The findings of the questionnaire study indicated that a considerable portion of households, approximately 72%, possessed knowledge about waste separation. However, despite being aware of this concept, they did not translate their knowledge into practical implementation. Conversely, only 28% of the respondents indicated actively applying their knowledge to properly separate waste at the household level.”

“Several waste types have been studied in some studies, encompassing organic waste, plastics, paper and paper products, metals, glass, rubber and leather, textiles, dirt and construction debris, hazardous wastes, and other forms of waste [9,21**]**. Notably, the classification of municipal solid wastes was expanded by Dangi et al. [11] and Dangi et al. [23] to include two new waste types, namely hazardous wastes, and dirt and building debris.”

“By categorizing their solid waste into several categories for collection and treatment, and encouraging the 3Rs (reduce, reuse, recycle), households can significantly improve waste management. A three-bin system, which allows residents to divide waste into dry recyclables, moist organic and/or domestic waste, and hazardous or residual waste, has been implemented in several places [9]. In Nepal, initiatives to encourage trash segregation have been gaining momentum. However, insufficient infrastructure, a lack of understanding, and restricted access to waste management services are frequently blamed for poor segregation practices. To improve waste segregation practices, it is essential to raise public awareness of its advantages, educate people about it, encourage public engagement, build segregation facilities, and develop the waste management system.”

**Household SWM practices**

We have refreshed as “The field survey indicated that solid waste collection had been effectively implemented in wards 1, 2, 3, 7, 8, and 9, respectively.”

We have replaced “ The composition of household solid waste generated per day in BM and the outcomes are summarized in Fig. 3” with “Assessment of the household waste composition generated in each ward of the Besisahar municipality is given in Table 5.”

We have added “The waste management study draws attention to the significant diversity in the Median, AVEDEV, and STDEV values. There are various levels of deviation from the mean in waste management practices in BM, as indicated by the AVEDEV range of 0.293 to 196.82. The range of median values, from 0.23 to 372, highlights the variety of results across waste management strategies. The STDEV values range from 0.375 to 250.9, revealing a wide range of practices with various degrees of consistency. In general, a normal distribution of waste generation has coefficients of skewness and kurtosis of 0 and 3, respectively [11, 24, 25]. An asymmetric distribution is measured by the skewness coefficient. Perfect symmetry is indicated by a coefficient of zero, while positive and negative skewness suggests a longer right tail and left tail. Our values for skewness range from 0.477 to 1.64, the distribution is moderately skewed and is approximately symmetric. It shows that all waste types had a positive skewness (to the right). The kurtosis coefficient measures the peakedness or flatness of a distribution. The calculated values of kurtosis range from -0.195 to 3.28, where positive kurtosis indicates heavier tails and the presence of outliers, while negative kurtosis suggests lighter tails and fewer outliers. Overall values of both coefficients showed the normal distribution of waste generation in BM.”

**Households waste characterization**

We have removed this “Over the past few years, there has been an increase in the amount of plastic waste.”

We have refreshed this text “The composition of household waste in Jeetpur Simara Sub-Metropolitan City was estimated to be 80% organic, 10% plastic, 6% paper, and 4% inert [21]. Among 53 municipalities in Nepal, organic waste had the highest household waste composition at 66%, followed by plastics at 12%, and paper products and paper waste at 9% [9]. An investigation conducted in 1995 found that only 38% of the farmers buried or burned old or unused pesticides, whereas others were disposed of in waste containers as solid waste in open fields [10].” and “ It was found that more than 70% of the municipal solid waste fraction is organic in nature (Pokhrel and Viraraghavan, 2005). ADB (2013) also reported that organic waste accounts for 56% of the total waste generated, excluding agricultural waste. In the context of Pokhara Metropolitan City, organic wastes comprise the largest fraction (73%) of the total waste generated, followed by inorganic waste (24%) and other wastes (3%). Similarly, in Janakpurdham Sub-Metropolitan City, organic wastes constitute the largest fraction of the total waste generated, followed by inorganic waste and other wastes (GoN, 2022). Das et al. (2018) also reported similar findings for waste composition in Pokhara Metropolitan City.“;

“Studies have consistently shown that a significant proportion of municipal solid waste is organic in nature. The composition of household waste in Jeetpur Simara Sub-Metropolitan City was estimated to be 80% organic, 10% plastic, 6% paper, and 4% inert [22]. Among 53 municipalities in Nepal, organic waste had the highest household waste composition at 66%, followed by plastics at 12%, and paper products and paper waste at 9% [9]. Pokhrel and Viraraghavan [1] found that more than 70% of the waste fraction falls into this category. ADB [9] reported that organic waste accounts for 56% of the total waste generated, excluding agricultural waste. In the specific context of Pokhara Metropolitan City, it was found that organic waste makes up the largest fraction, constituting 73% of the total waste generated, followed by inorganic waste at 24%, and other wastes at 3%. Similar patterns were observed in Janakpurdham Sub-Metropolitan City, where organic waste comprised the largest fraction, followed by inorganic waste and other wastes [20]. Even though the high level of food percentage in Nepal, is still lower than in Iran (Rasht city) with a value of 85% [17]. Our findings align with the waste composition results reported by Das et al. [26] for Pokhara Metropolitan City.”

We have added the following new topic in the same section’ “**Pearson correlation matrix between population and various wastes”**

The Pearson correlation matrix is a useful tool for analyzing relationships in the field of solid waste management. Researchers and waste management professionals can build efficient waste management strategies by understanding the relationships between population and other waste parameters. Understanding these relationships enhances decision-making and improves waste management approaches.

The researchers have been employed a Pearson’s correlation matrix to explore the degree of association among various factors in waste management practices. The correlation values, ranging from 0.7 to 1, indicate a strong correlation, while values between 0.5 and 0.7 suggest a moderate correlation [27]. Table 6 presents the Pearson correlation coefficients for different solid waste management (SWM) parameters in each ward of BM. The analysis revealed significant associations within the context of solid waste management.

Population was found to be strongly correlated with a number of waste types, including organic (r=0.798), paper (r=0.851), plastics (r=0.793), metals (r=0.779), textile (r=0.743), glass (r=0.810), rubber (r=0.845), construction materials (r=0.770), pharmaceutical (r=0.818), electronic (r=0.835), and other wastes (r=0.826). These results indicate that the generation of organic, paper, plastics, metal, textile, glass, rubber, and construction waste consistently increases with population, highlighting strong positive associations across all parameters. These relationships offer valuable perspectives on influencing factors, enabling waste management practitioners and policymakers to make well-informed decisions.

On the other hand, agricultural waste exhibited weak correlations (r=0.141, 0.038, and 0.004) with population, paper, and textile, respectively. These specific values point to the absence of a substantial correlation and suggest that different variables may be influencing different parameters. Moreover, agricultural waste displayed a negative correlation with organic (r=-0.048), plastic (r=-0.183), metals (r=-0.260), glass (r=-0.023), rubber (r=-0.151), construction materials (r=-0.219), pharmaceutical (r=-0.161), electronic (r=-0.210), and other wastes (r=-0.020). Notably, an increase in population was found to be associated with a decrease in agricultural waste. This negative correlation between certain waste types and agricultural waste suggests a trade-off relationship, where an increase in one variable corresponds to a decrease in the other to a certain degree.

Table 6. Pearson’s correlation matrix for population and SW in different wards of BM (r: range = -1.00 to +1.00).

|  | Population | Organic | Paper | Plastics | Metals | Textile | Glass | Rubber | Agricultural | Construction | Pharmaceutical | Electronic | Others |
| --- | --- | --- | --- | --- | --- | --- | --- | --- | --- | --- | --- | --- | --- |
| Population | 1 |  |  |  |  |  |  |  |  |  |  |  |  |
| Organic | 0.798 | 1 |  |  |  |  |  |  |  |  |  |  |  |
| Paper | 0.851 | 0.967 | 1 |  |  |  |  |  |  |  |  |  |  |
| Plastics | 0.793 | 0.927 | 0.911 | 1 |  |  |  |  |  |  |  |  |  |
| Metals | 0.779 | 0.855 | 0.861 | 0.973 | 1 |  |  |  |  |  |  |  |  |
| Textile | 0.743 | 0.944 | 0.924 | 0.926 | 0.84 | 1 |  |  |  |  |  |  |  |
| Glass | 0.81 | 0.952 | 0.977 | 0.91 | 0.873 | 0.938 | 1 |  |  |  |  |  |  |
| Rubber | 0.845 | 0.942 | 0.942 | 0.884 | 0.851 | 0.897 | 0.964 | 1 |  |  |  |  |  |
| Agricultural | 0.141 | -0.048 | 0.038 | -0.183 | -0.26 | 0.004 | -0.023 | -0.151 | 1 |  |  |  |  |
| Construction | 0.77 | 0.965 | 0.925 | 0.922 | 0.852 | 0.939 | 0.921 | 0.954 | -0.219 | 1 |  |  |  |
| Pharmaceutical | 0.818 | 0.884 | 0.856 | 0.819 | 0.736 | 0.86 | 0.835 | 0.918 | -0.161 | 0.946 | 1 |  |  |
| Electronic | 0.835 | 0.951 | 0.933 | 0.926 | 0.878 | 0.926 | 0.941 | 0.982 | -0.21 | 0.986 | 0.952 | 1 |  |
| Others | 0.826 | 0.987 | 0.987 | 0.94 | 0.875 | 0.956 | 0.97 | 0.944 | -0.02 | 0.956 | 0.889 | 0.953 | 1 |

**Household waste generation**

We have added “In Waling municipality, there were 5,952 households and an estimated population of 51,234 individuals. The average per capita household waste generation rate in Waling was 0.68 kg/person/day, leading to a total daily household waste generation of approximately 5.51 tons. It is noteworthy that household waste comprises a significant portion, accounting for approximately 50% to 75% of the overall MSW [9].”

We have also added the following new topic in the “Results and discussion” part;

**Total quantity of waste generated in BM**

Waste generation in a municipality is influenced by various factors. Among them, the size and density of the population are crucial determinants, as larger urban areas typically produce more waste [11]. Economic development is another significant factor, as it leads to increased production of goods and services, consequently resulting in higher waste generation. Moreover, consumer behaviour and lifestyle choices have a substantial impact on the types and quantities of waste produced. Societies that heavily rely on packaged goods and single-use products tend to generate more waste compared to those practising sustainable consumption [1, 9, 11]. These factors collectively shape the waste generation patterns in a given municipality.

In Besisahar municipality, solid waste generation was determined by considering several factors into account. The average per capita household waste generation was determined by multiplying the average waste generation by the total number of residents, resulting in an estimated 7427.75 kg/household/day. This household waste constituted approximately 46.02% of the total waste generated in the municipality. Apart from households, other sectors also contributed to waste generation such as hospitals generated 124.96 kg/day (accounting for 0.77% of the total waste), hotels and restaurants contributed 3,270 kg/day (20.28%), commercial shops accounted for 5,000 kg/day (30.98%), academic institutions generated 116.9 kg/day (0.72%), banks and financial institutions produced 34.4 kg/day (0.21%), government and private offices generated 64 kg/day (0.39%), and industries contributed 100 kg/day (0.61%). Collectively, all these sectors contributed to approximately 16138.01 kg/day in the entire Besisahar municipality.

In Bhimeshwor Municipality, the total daily waste generation was estimated to be around 15,011 kg/day, which aligns with the findings of the waste analysis conducted in this municipality. Likewise, Janakpurdham Sub-Metropolitan City generated a total waste of 78,273 kg/day, consistent with the waste analysis in Bhimeshwor Municipality and our study area. In Pokhara Metropolitan City, the total daily waste generation amounted to 134,500 kg/day. This was composed of 94,150 kg/day (70%) from households, 6,725 kg/day (5%) from businesses, 6,725 kg/day (5%) from industries, 13,450 kg/day (10%) from educational institutions, 4,559.6 kg/day (3%) from health institutions, and 8,890.4 kg/day (7%) from other sectors. On an annual basis, the average total waste collected per municipality in the years 2073/74, 2074/75, and 2075/76 was approximately 2,231 metric tonnes (mt), 2,164 mt, and 2,233 mt, respectively, according to data from the Government of Nepal. This translates to an average daily waste collection per municipality of 6.1 mt, 5.9 mt, and 6.1 mt, respectively, over the course of those three years [20].

Our findings indicate that waste generation per capita per day is higher in wards located in city areas, attributed to factors such as higher population density, increased economic activities, and different consumer behaviors prevalent in urban areas. Indeed, the effectiveness of waste management systems and practices implemented by a municipality can significantly influence the total quantity of waste generated [1]. To address this issue, implementing efficient waste management strategies, such as recycling, composting, and waste reduction initiatives can play a significant role in minimizing waste generation [11]. Additionally, the regulatory framework and policies established by local governments are vital in promoting sustainable waste management practices. Legislation mandating waste segregation, promoting recycling, and imposing waste disposal fees can serve as incentives for residents and businesses to adopt more environmentally friendly practices, leading to a reduction in waste generation [9]. By considering these factors and implementing appropriate measures, municipalities can work towards improving waste management and achieving a reduction in overall waste generation, contributing to a more sustainable and cleaner environment.

**Practices of SWM in urban areas**

We have added the following in the last two paragraphs;

In the Kathmandu Valley, it has been estimated that approximately 3% (20 tons/day) of the municipal solid waste, is disposed of through open burning. This releases harmful particulate matter, gaseous pollutants, and toxic substances into the atmosphere, contributing to air pollution and environmental degradation [26]. Similarly, in Bhimeshwor Municipality, approximately 53% (7,951 kg/day) of the waste generated is collected and disposed of in landfills [20]. Proper landfill management is important to prevent environmental contamination and potential health hazards associated with improper waste disposal. These figures underscore the significance of adopting effective waste management practices, such as waste reduction, recycling, and sustainable disposal methods.

The combination of biodegradable and non-biodegradable wastes leads to the production of methane gas during anaerobic digestion and the release of harmful substances from waste dumps, which can contaminate groundwater resources. To address this issue, specific recyclable materials such as plastic containers and packaging, cans, glass and PET bottles, used paper, clothes, and oversized waste items need to be collected separately for reuse or recycling purposes, while non-recyclable waste, on the other hand, should undergo incineration. Unfortunately, many landfill sites in Nepal fail to meet scientific or health standards, and waste is not treated prior to disposal, resulting in the creation of hazardous dumpsites [9].

**Response to the current waste disposal system**

“Questionnaires were given randomly to the heads of households in rural and urban areas regarding the waste disposal in BM, and the results obtained from this survey are listed in Fig. 9.” has replaced by “Table. 10 shows statistical parameters for response regarding waste disposal in BM.”

**Linkages with SDGs**

We have added the following in the last paragraphs.

“Effective household SWM practices play a vital role in aligning with SDGs 11, "sustainable cities and communities," 12, "ensuring sustainable consumption and production patterns, and 14, "Life below water". These practices contribute significantly to environmental protection, particularly at the local and municipal levels. However, several regions in Asia are currently facing challenges in meeting Target 11.6, which focuses on improving waste management in urban areas [46]. Since SWM policies and services are typically under the responsibility of local governments, enhanced support at the municipal level can exert pressure on higher levels of government, compelling them to take action and allocate additional funding. This proactive approach directly addresses one of the major barriers to progress in waste management.”

“Hence, local policymakers must acknowledge the interdependence of SWM targets and other SDGs. Recognizing that advancements in WM can drive progress in other SDGs, and vice versa, is essential. This understanding enables local policymakers to advocate for comprehensive WM strategies that not only benefit the environment but also contribute to broader sustainable development goals. However, to achieve these objectives, significant investments are necessary. Adequate funding is required to address the lack of robust SWM systems and to support educational campaigns emphasizing the importance of waste separation. Without sufficient financial commitments, inadequate waste management practices will persist, impeding sustainable development efforts, especially in rapidly expanding urban areas across Asia.”

**Conclusions and future recommendations**

We have added as “The results obtained from Pearson correlation coefficients indicated robust correlations among the parameters of different types of wastes generated in BM.”

Thanks for the reviewers’ suggestion.

6. **Lack of proper statistical analysis. Authors should perform some advanced statistical analysis for figures 3, 4, and 9. A description of statistical techniques and tests applied to the analyses of data sets must be mentioned.**

**Response**: We have calculated the statistical parameters for Fig. 2 and now this figure is replaced by the following Table.

**Fig. 2.**

**Table 5.** Assessment of the household waste composition generated in each ward of the Besisahar municipality.

| Types of waste | Statistical parameters | | | | | | | | | |
| --- | --- | --- | --- | --- | --- | --- | --- | --- | --- | --- |
|  | Min and max waste generation BM’s wards | | Mean (Kg/day) | Total waste in BM (Kg/day) | % | Median | AVEDEV | STDEV | Skewness | Kurtosis |
|  | Minimum (Kg/day) | Maximum (Kg/day) |  |  |  |  |  |  |  |  |
| Organic | 125 (4)^*^ | 912 (7)^*^ | 449.82 | 4948.02 | 68.03 | 372 | 196.82 | 250.9 | 0.788 | -0.196 |
| Paper | 16.25 (5)^*^ | 139 (8)^*^ | 58.04 | 638.5 | 8.13 | 45.1 | 29.85 | 39.42 | 0.941 | 0.707 |
| Agricultural | 27.35 (3)^*^ | 62.89 (11)^*^ | 38.31 | 421.44 | 5.5 | 34.07 | 7.62 | 10.44 | 1.32 | 2.23 |
| Plastics | 9.3 (4)^*^ | 80 (8)^*^ | 9.64 | 412.38 | 5.21 | 37.48 | 17.55 | 23.63 | 0.477 | -0.676 |
| Construction | 7.13 (5)^*^ | 72.14 (8)^*^ | 26.72 | 293.95 | 3.81 | 17.1 | 18.54 | 22.87 | 1.11 | 0.201 |
| Textile | 7.5 (5)^*^ | 41 (7)^*^ | 19.5 | 214.5 | 2.72 | 12 | 11.72 | 13.47 | 0.736 | -1.23 |
| Metals | 1.26 (5)^*^ | 8.51 (7)^*^ | 3.89 | 42.84 | 0.54 | 3.79 | 31.71 | 2.24 | 0.586 | 0.255 |
| Glass | 3.32 (3)^*^ | 19 (7)^*^ | 7.25 | 79.84 | 1.01 | 5.1 | 3.90 | 5.04 | 1.32 | 1.81 |
| Rubber | 0.13 (4)^*^ | 3.01 (7)^*^ | 0.782 | 8.61 | 0.109 | 0.26 | 0.782 | 1.02 | 1.45 | 1.57 |
| Electronic | 0102 (5)^*^ | 1.12 (8)^*^ | 0.391 | 4.30 | 0.055 | 0.23 | 0.293 | 0.375 | 1.25 | 0.728 |
| Pharmaceutical | 0.12 (3)^*^ | 3.08 (8)^*^ | 0.761 | 8.374 | 0.108 | 0.35 | 0.67 | 0.937 | 1.64 | 3.28 |
| Others | 14 (4)^*^ | 64 (7)^*^ | 32.27 | 355 | 4.78 | 28 | 13.07 | 16.59 | 0.817 | -0.195 |

**Note:** *: Total waste generated in respective ward number.

**Response:** Figure 3 shows only the percentage composition and the statistical parameters of households for g/capita/day. We have now compared our statistical results with others. Please see the following Table.

Table 7. Comparison of statistical parameters for household waste generation (g/capita/day) in Nepal.

| Study area | Statistical parameters | | | | | | | | | Reference |
| --- | --- | --- | --- | --- | --- | --- | --- | --- | --- | --- |
|  | Sample size | Minimum (g/capita/day) | Maximum (g/capita/day) | Average (g/capita/day) | Median | AVEDEV | STDEV | Skewness | Kurtosis |  |
| Besisahar Municipality | 230 | 1.64 | 9110 | 197.60 | 10.04 | 318.84 | 923.68 | 7.29 | 58.35 | Present study |
| Kathmandu Metropolitan City | 333 | - | - | 497.3 | - | - | 5349.4 | 18.2 | 332.1 | Dangi et al., 2011 |
| Tulsipur Municipality | 84 | 2.8 | 12033.3 | 330.4 | - | - | 1317.6 | 8.7 | 77.5 | Dangi et al., 2013 |

**Response:** Figure 9 shows the response for SWM practices in Besisahar municipality. We have also calculated the statistical parameters and this figure is replaced by the following Table..

**Fig. 9**

**Table. 8.** Statistical parameters for response regarding waste disposal in BM.

| Questionnaire | No. of participants (n=230) | | | | | | | | | | Median | AVEDEV | STDEV |
| --- | --- | --- | --- | --- | --- | --- | --- | --- | --- | --- | --- | --- | --- |
|  | Strongly agree | % | Agree | % | Strongly disagree | % | Disagree | % | No knowledge | % |  |  |  |
| Suitable for dumping area on riverbank | 6 | 2.60 | 18 | 7.82 | 57 | 24.78 | 138 | 60 | 11 | 4.78 | 18 | 41.2 | 55.21 |
| Impact of dumping on river banks | 48 | 20.86 | 110 | 47.82 | 22 | 9.56 | 34 | 14.78 | 16 | 6.95 | 34 | 26.4 | 37.8 |
| Segregation of waste before dumping | 58 | 25.21 | 115 | 50 | 14 | 6.08 | 18 | 7.82 | 25 | 10.86 | 25 | 32.4 | 42.29 |
| Waste treatment before dumping | 35 | 15.21 | 92 | 40 | 23 | 10 | 46 | 20 | 34 | 14.78 | 35 | 18.4 | 26.97 |
| Private waste collector activities on dumping | 19 | 8.26 | 94 | 40.86 | 34 | 14.78 | 69 | 30 | 14 | 6.08 | 34 | 28.4 | 34.38 |
| Municipality in waste disposal activities | 28 | 12.17 | 76 | 33.04 | 46 | 20 | 69 | 30 | 11 | 4.78 | 46 | 21.2 | 27.28 |
| Implementation of SDGs | 2 | 0.86 | 7 | 3.04 | 46 | 20 | 83 | 36.08 | 92 | 40 | 46 | 33.2 | 41.65 |

7. **The visibility of all supplied figures (Fig. 6, 7, and 9) is not good. Authors are advised to provide the figures in high resolution with good font visibility. The size of the fonts is very small, please increase it also.**

**Response:** Fig 9 is replaced by Table 8 and the following figures 6 and 7 are given in the revised manuscript.

**Fig. 5.**

**Fig. 5.**

**In conclusion,**

We have added: “The results obtained from Pearson correlation coefficients indicated robust correlations among the parameters of different types of wastes generated in BM."

We have incorporated all the recommended changes based on the reviewers' comments, and we are hopeful that this revised manuscript is now suitable for publication in the PLoS ONE journal.
